# Sociotechnical Cybersecurity Framework for Securing Health Care From Vulnerabilities and Cyberattacks: Scoping Review

**DOI:** 10.2196/75584

**Published:** 2025-10-15

**Authors:** Pius Ewoh, Tero Vartiainen, Timo Mantere

**Affiliations:** 1 School of Technology and Innovations Information Systems Science University of Vaasa Vaasa Finland; 2 School of Technology and Innovations Automation Technology University of Vaasa Vaasa Finland

**Keywords:** computer security, network security, digital health, health information, electronic health record system, cyber threats, ransomware, breaches

## Abstract

**Background:**

The vulnerability of health care systems to cyberattacks and breaches of health information is on the rise worldwide. Considering the increasing rate of reported cyber incidents and the risks they pose to patient safety, privacy, and financial losses, there is a need to examine the way cybersecurity is conceptualized in health care organizations, taking into account technology, processes, and humans.

**Objective:**

This study examined the dynamics of the factors of vulnerabilities and cyberattacks in the context of sociotechnical systems theory underlying the relationships among humans, technology, and processes. It developed a conceptual sociotechnical cybersecurity framework for preventing vulnerabilities and responding to cyberattacks and threats in health care systems.

**Methods:**

A scoping review was conducted to search the extant literature in 3 databases—Web of Science, PubMed (MEDLINE), and Scopus. A total of 1375 papers from the period of 2012-2024 were retrieved, 76 of which, in the domain of health care and cybersecurity, were reviewed and analyzed. Original research and review papers were included. Only published English-language papers were included to focus on contemporary issues, challenges, and solutions. Relevant information from the included sources was charted and summarized. The study characteristics were extracted from the included papers, and the evidence was synthesized using thematic analysis.

**Results:**

Of the 1375 papers identified, 76 (5.5%) met the inclusion criteria. The results showed that the factors of vulnerabilities to cyberattacks comprise 12 subfactors in health care systems. Concerning technology-related factors of vulnerabilities, most studies described the complex system design and usability (16/76, 21%) and integration of new technology (15/76, 20%) as challenges in health care systems. Concerning human-related factors, most studies described a shortage of skilled professionals and limited budgets as contributing to poor cybersecurity management. The study found that processes involved both technology and humans relative to the unit factors of vulnerabilities to cyberattacks. There was a sociotechnical interplay across the factors of vulnerabilities. The concept of sociotechnical cybersecurity offers a comprehensive and explicit perspective on the sociotechnical underpinning and joint optimization required to advance cybersecurity toward achieving sustainable health care systems.

**Conclusions:**

The conceptual framework of sociotechnical cybersecurity provides a contemporary foundation and deep insight for identifying and preventing vulnerabilities and responding to cyberattacks in health care systems. The framework is important due to its suitability, applicability, and customizability for dynamic and complex health care systems. The study also provides compliance standards for applying the proposed conceptual framework to guide health care organizations in cybersecurity practices. The study of cybersecurity through the sociotechnical lens in the health care domain is limited. Further studies are needed on cybersecurity incident management. Health care organizations should leverage the strength of cybersecurity through the implementation of risk assessment and incident response plans.

## Introduction

### Background

The digitalization of the health care system has introduced numerous positive effects and gains, such as easy access to health information and effective and efficient health care delivery processes and outcomes [1]. In the last 2 decades, health care digitalization has emerged as a topic of discussion among stakeholders in securing critical infrastructure. Understanding how health care professionals use digital technologies to provide high-quality care requires a stakeholder’s viewpoint.

Technology integration is the implementation of electronic health records (EHRs), integration of Internet of Medical Things (IoMTs) devices, and broader IT infrastructure. The rapid integration of these technologies into health care systems created this pathway of improved access to medical services, enhanced patient outcomes, and streamlined workflows for health care providers and services in a borderless, continuous health care journey for transitional nations. Patient health care diagnostics reports and information can be accessed in real-time to enable managing medical history and response to emergency cases with the use of EHR systems. However, this has introduced significant vulnerabilities, making health care systems more susceptible to cyberattacks that could compromise sensitive patient data and disrupt health care services [1-3]. As these vulnerabilities are linked to their areas of occurrence, they can be categorized and described through the interplay of technology, humans, and processes. This enables the application of sociotechnical systems (STS) theory and knowledge management approaches to health care systems [4,5]. The National Institute of Standards and Technology (NIST) Cybersecurity Framework acknowledges that these vulnerabilities may arise from human factors, technology, and organizational processes [6]. Additionally, the research by Kaberuka and Johnson [7] on adapting the STAMP (Systems Theoretic Accident Model and Processes) for sociotechnical cybersecurity challenges in emerging nations acknowledges that human factors, organizational processes, and technology are of great concern. These vulnerabilities must be addressed for organizations to maintain resilience to cyberattacks and threats. NIST interagency and internal reports define these vulnerabilities as weaknesses in an information system, system security procedures, internal controls, or implementation that could be exploited or triggered by a threat source [8,9].

The relationships among technology, humans, and organizational processes lead to vulnerabilities exploited by cybercriminals or state-sponsored attackers to gain access and control over critical health care infrastructure and sensitive data, thereby disrupting health services. These vulnerabilities can be considered a sociotechnical problem in a complex health care system [7,10-12]. This problem can be solved using a sociotechnical approach to tackling vulnerabilities in health care systems. According to the 2024 report of the World Economic Forum, the cost of damage incurred by all forms of cybercrime resulting from humans, technology, and organizational processes could reach US $10.5 trillion in 2025. Some of the main sociotechnical cybersecurity problems in health care systems include the following. First, in 2021, ransomware attacks were launched on the health care systems of Ireland, known as the Health Service Executive, disrupting the health care services of 54 public hospitals, and IT systems nationwide were shut down. As a result, more than 80% of the IT environment was encrypted by cybercriminals, and information was exposed at a great financial cost [13,14]. Second, the WannaCry ransomware attacks in 2017 infected over 200,000 computers worldwide and disrupted services due to vulnerabilities in computer operating systems [15-17]. Third, in 2017, Hollywood Presbyterian Medical Center was also attacked by ransomware that encrypted all health information. The medical center paid a ransom of US $17,000 to regain access to its data [18]. Fourth, in 2016, Lukaskrankenhaus, a public hospital in Germany, was attacked by ransomware initiated through phishing. Computer systems were forced by authorities to shut down [19].

Based on this knowledge gap identified, the following research questions (RQs) were asked: (1) What are the sociotechnical factors of vulnerabilities to cyberattacks that affect health care systems? (RQ 1) (2) What kind of framework is best suited for preventing vulnerabilities and responding to cyberattacks and threats in health care systems? (RQ 2). The objective of this study was to examine the dynamics of the factors of vulnerabilities to cyberattacks from a sociotechnical perspective and develop a conceptual framework for preventing vulnerabilities and responding to cyberattacks and threats in health care systems.

### Rationale and Sociotechnical Perspective

#### Rationale

The motivation for this research emerged following the increasing number of cyberattacks in health care organizations. Preventing cyberattacks requires an understanding of the multidimensional complexities of health care system factors of vulnerabilities. However, few studies have been conducted in the field of cybersecurity in health care from a sociotechnical perspective. Garcia-Perez et al [20], Szczepaniuk and Szczepaniuk [21], and Vukotich [22] addressed cybersecurity challenges in health care systems from a technical perspective. Zimmermann and Renaud [23] and Nicho and McDermott [24] focused on addressing vulnerabilities in health care organizations using a social approach. This contributes to the literature by addressing the scholarly call for a sociotechnical cybersecurity framework in health care aimed at preventing vulnerabilities and responding to cyberattacks and threats [25-27]. Nicho and McDermott [24], Wani et al [28], and Sutton and Tompson [29] noted that a comprehensive cybersecurity framework that closes the sociotechnical gap within health care organizations’ cyberspace is important. A study conducted by Malatji et al [17] found that “only four security frameworks, namely NIST, ISO/IEC, COBIT, and IT-CMF partially fulfilled the security requirements of the social dimension of a sociotechnical system” [25].

Scholars have contributed to cybersecurity theory by developing various generic frameworks for different types of organizations [17,29-31]. This study proposed a conceptual sociotechnical cybersecurity framework for health care organizations to prevent vulnerabilities and respond to cyberattacks.

#### STSs Perspective

The STS theory examines the introduction of new technologies in organizations, their impact on humans, and the interactions between individuals of different skill sets, all within organized units to optimize the performance of social and technical systems [32,33]. According to Trist [33], an STS perspective in any organization comprises a set of integrated and interacting social and technical subsystems or constructs, such as people, infrastructure, technology, culture, goals, and processes. At their core, STSs conceptualize the design and performance of any organizational system that can only be optimized if there is an integration and interplay of the social and technical aspects, and they are deemed interdependent parts of a complex system.

The term STSs originated with Emery and Tris in 1960, as they observed that systems involve complex interactions among people, machines, and the environmental aspects of the organizational system [34]. The concept of STS theory was proposed by the Tavistock Institute as a method used to treat wounded soldiers and in constructions by Mumford [35], Emery [36], and Trist [37]. The underlying assumption of STSs advocates that systems design should be a process that considers both social and technical aspects that influence the functionality and usage of interconnected computer-based systems [38].

**Figure 1:**
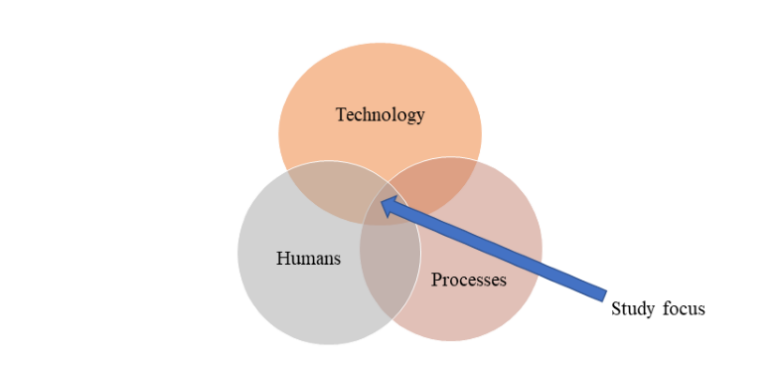
Sociotechnical interplay.

This study adopted an STS perspective on cybersecurity in the domain of health care that integrates technology, humans, and processes, subsystems, or constructs. In the context of cybersecurity in health care, the aforementioned constructs were established in the study conducted by Zimmermann and Renaud [23]. Figure 1 illustrates the 3 areas of STSs that were integrated in a holistic approach to prevent vulnerabilities and respond to cyberattacks in health care systems through an intervention framework [16,25,27].

## Methods

### Protocol and Registration

The review was performed based on the PRISMA-ScR (Preferred Reporting Items for Systematic Reviews and Meta-Analyses extension for Scoping Reviews) checklist by the JBI (Joanna Briggs Institute) [39,40]. This study aimed to examine the dynamics of the factors of vulnerabilities to cyberattacks and propose a conceptual framework for health care systems. During the planning stage of this scoping review, a protocol was created that reflected sources of information, search strategies, inclusion and exclusion criteria, source selection, and data charting processes. This scoping review protocol was not registered. The PRISMA-ScR checklist is presented in Multimedia Appendix 1.

### Information Sources

Three scientific databases—Web of Science, PubMed (MEDLINE), and Scopus—were searched to retrieve relevant papers, including both original research and review papers.

### Search

Search queries were customized to the syntax and indexing features of each database. Keyword searches targeted the key concepts of cyberattacks and health care for PubMed, Scopus, and Web of Science. The title and additional abstract search terms were used to identify relevant publications. Truncation was used to identify word variations of the key concepts in different publications. The search terms were separated with the Boolean operators “AND” and “OR.”

PubMed (MEDLINE) incorporated a combination of Medical Subject Headings, including computer security, health care facilities, workforce, services, and delivery of health care. An example of the search strategy in one of the databases is shown in Textbox 1. The detailed search strategy used for the other databases is provided in Multimedia Appendix 2.

**Search strategy showing the search string for PubMed.**

“Computer Security”[Mesh] OR Cyberattack*[tw] OR Cybercrime*[tw] OR “Cyber Crime”[tw] OR Cyberthreat*[tw] OR “Cyber Threat”[tw] OR “Cyber Crises”[tw] OR “Cyber Risk”[tw] OR “Cyber Incident”[tw] OR Cyber Operation[tw] OR Cyberspace[tw] OR “Cyber Infrastructure”[tw] OR “Data Breach”[tw] OR “Data Security”[tw] OR “Firewall”[tw] OR “Information Security”[tw] OR “Information Technology Security”[tw] OR “Information Systems Security”[tw] OR “Security Incident”[tw] OR “Network Security”[tw] OR Ransomware[tw] OR Malware[tw] OR Phishing[tw] ) AND ( “Health Care Facilities, Workforce, and Services”[Mesh] OR “Delivery of Health Care, Integrated”[Mesh] OR “Health Care”[tw] OR “Health Information”[tw] OR “Health Information Management”[tw] OR “Healthcare Systems”[tw] OR “Health Systems”[tw] OR “Health System Infrastructure”[tw] OR “Medical Devices”[tw] OR Medical Technolog*[tw] OR Health Technolog*[tw] OR Health Care Technolog*[tw].

### Eligibility Criteria

The inclusion criteria for the papers were relevance to health care cybersecurity, coverage of cybersecurity issues, challenges, and solutions in health care systems. Only English-language papers published between 2012 and 2024 were included (Table 1).

**Table 1:** Inclusion and exclusion criteria.

Criterion	Inclusion	Exclusion
Language of papers	Papers in English	Non–English-language papers
Year of publication	Papers published between 2012 and 2024	Papers published outside the range of 2012-2024
Research topic focus	Cybersecurity and health care	The topic is different from the topic areas
Scope of work	Key elements and factors that contribute to or lead to breaches, cyberthreats, cyberattacks, and vulnerabilities, and the development of a sociotechnical intervention framework for health care system resilience	Topics outside the research scope of work
Publication type	Original research and review papers	Research in-progress papers, editorial papers, and theses

### Selection of Sources of Evidence

The retrieved papers were exported to the citation tool Zotero (Digital Scholar), in which duplicates were identified and removed using the duplicate item function. To assess eligibility, the titles and abstracts of each paper were analyzed by 2 of the authors. In instances in which the eligibility criteria for the papers were not clear, all 3 authors checked the papers and perused them to assess their relevance.

### Data Charting Process

Using a standard Microsoft Excel (Microsoft Corp) spreadsheet, data from the studies that met the eligibility criteria were extracted independently by one of the authors and assessed by the other 2 authors to ensure data quality and consistency. This was used to identify the key characteristics of each study and relevant information regarding cyberattacks in health care.

### Data Items

The key data items extracted included author, year of publication, country of origin, study design, aims, and key findings. The extracted data items were checked by the second author. A list of the extracted characteristics for the included studies (N=76) is provided in Multimedia Appendix 3 [1,4,6,7,9,11,12,16-18,20-23,25-27,41-99].

### Critical Appraisal Within Sources of Evidence

The quality of the source of evidence was checked by 2 authors using 3 different appraisal tools. Joanna Briggs Critical Appraisal Tools were used for qualitative research [100], the Mixed Methods Appraisal Tool [101] was used for mixed methods studies, and the Centre for Evidence-Based Medicine Critical Appraisal Checklist was used for cross-sectional studies [102] and the Scale for the Assessment of Narrative Review Articles Appraisal Tool for narrative review papers [103]. This was carried out to ensure that the sources of evidence were up-to-date, relevant, and reputable. For instances in which this was not clear, all 3 authors assessed the sources (Multimedia Appendix 4 [1,4,6,7,9,11,12,16-18,20-23,25-27,41-99,104]). However, the JBI Manual for Evidence Synthesis suggests that critical appraisal is not required for scoping review [40,105]. Multimedia Appendix 5 elucidates the different quality appraisal methods in detail. Studies were not excluded based on quality to capture as much literature as possible; however, low-quality studies were not used to draw conclusions.

### Synthesis of Results

Thematic analysis was conducted manually following the 6-step approach described by Braun and Clarke [106]. The 6-step approach involves familiarization with data, generating initial code by using sticky notes, searching for themes, reviewing the themes, defining and naming the themes, and producing the report. The analysis is hybrid in nature. The results were presented for the data extracted from the relevant papers in tabular form and descriptive formats (categorized into themes), which aligned with the objective and scope of the review.

## Results

### Overview

**Figure 2:**
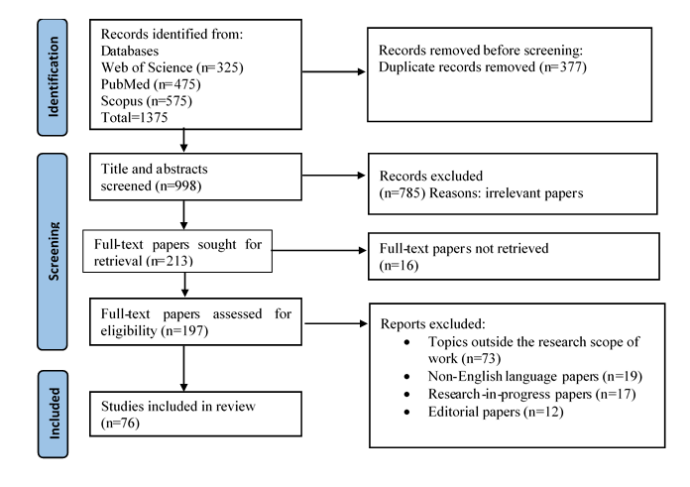
PRISMA diagram for paper selection. PRISMA: Preferred Reporting Items for Systematic Reviews and Meta-Analyses.

A total of 1375 papers were identified from the databases. Thereafter, 377 duplicate papers were removed, and 998 were screened. Subsequently, 213 full-text papers underwent screening. In the end, 76 papers were included in the review (Figure 2 illustrates the selection process).

The review of the extant literature confirmed the 3 factors of vulnerabilities to cyberattacks (technology, humans, and processes) from the lens of the STS theory in health care systems; they are presented in Tables 2-4. These factors were further categorized into twelve subfactors: (1) new technology integration, (2) complex system design and usability, (3) third-party application and plugin, (4) limited monitoring, (5) inadequate access control management, (6) insider threats, (7) shortage of skilled professionals and limited budget, (8) inefficient training, (9) security culture, (10) untimely incidence response and recovery plan, (11) inadequate policy and procedure, and (12) lack of regular audit and assessment. Subsequently, these 12 subfactors were outlined in descriptive formats.

**Table 2:** Technology factors.

Technology			Studies, n (%)		References
**New technology integration**			15 (20)		
	New technology integration into health care systems creates a new landscape for health care systems to be vulnerable to cyberattacks and threats.			[9,11,20,41-45]	
	Inappropriate technology integration creates loopholes and interoperability and compatibility challenges that lead to cyberattacks and threats.			[1,27,46]	
	Interconnected medical and end point devices, when exposed to the internet, create security risks that are possible points of access for cyberattackers to gain access to health care systems.			[16,23,42,47,48]	
**Complex system design and usability**			16 (21)		
	Complex system design tends not to be user-friendly; thus, its application in health care systems creates ambiguity in managing cloud-based big data and information, which results in exploitation by cybercriminals.			[12,47,49,50]	
	Design limitations on implanted medical sensor devices, such as assembly size and limited energy source, lead to connectivity and communication interruption for health care professionals in monitoring patients and data due to denial-of-service attacks. Such limitations also create encryption challenges.			[44,51]	
	Lack of a comprehensive or holistic framework for the security design in all layers of connected medical devices and software applications creates health information and privacy risks for internet-based device architecture and the operational environment.			[21,26,52,53]	
	Highly complex interconnected network systems increase the likelihood of vulnerabilities.			[9,23,27,54,55,99]	
**Third-party applications and plugins**			7 (9)		
	Software internet-based products from third-party applications leverage vulnerabilities in medical devices and authentication errors that can be exploited by hackers to steal sensitive data or manipulate health care system operations.			[42,51]	
	Most incidents of vulnerability and cyberattacks in health care systems stem from a wide range of sources, such as operating systems or cloud-based software architectures of third-party developers.			[56-58]	
	Third-party universal applications and devices, such as mobile apps and hardware integration in health care systems used for telemedicine applications, are not able to provide user anonymity when confronted with cyberattacks.			[59,60]	
	Health care plugin apps for mobile devices often face privacy and security issues due to developer deviation from compliance with regulatory standards.			[42,61]	
**Limited monitoring**			11 (15)		
	Inadequate capabilities for continuous monitoring of systems result in health information breaches and cyberattacks in health care systems.			[4,12,22,61,90]	
	Inconsistent monitoring affects compliance, health care cyber-critical infrastructure updates, and organizational processes. This invariably constrains organizations’ preparedness to achieve the goals of security standards.			[52,65,83,91]	
	Complexities in monitoring processes in health care organizations are a gateway to data breaches, cyber threats, and cyberattacks.			[68,69]	
**Inadequate access control management**			8 (11)		
	Reactive health care systems that lack a strong access control system are prone to privilege escalation attacks.			[4,71,84,91]	
	In the course of a malware incident, attackers can modify access control systems to grant administrative privileges to exploit health care systems.			[46,84,92]	
	Breakdown in access control management resulting from an update, server disruption, or malicious intrusion pushes health care organizations to shut down operational processes in the event of a cyberattack to reduce harm.			[52,67,92]	

**Table 3:** Human factors.

Humans			Studies, n (%)		References
**Insider threats**			7 (9)		
	Insiders can introduce threats and vulnerabilities through inadvertent actions, such as inappropriate behavior, clicking phishing links, and falling victim to cyber threats.			[9,42,62]	
	Most of the breaches that occur in health care organizations originate with insiders stealing and leaking sensitive information to cybercriminals for money or political gain.			[9,11,52,63]	
	Negligence by internal IT teams in failing to terminate vendor accounts or agreements in intersupport systems of care could create an entry point for vulnerability to cyberattacks.			[52,61]	
**Inefficient training**			9 (12)		
	Health care cybersecurity training implementations are largely misdirected, with a focus on cybersecurity professionals and information and communication technology (ICT) departments only, while neglecting health care–based professionals.			[62,64,65]	
	Ineffective cybersecurity training helps cybercriminals gain access to a health care system’s sensitive information through social engineering methods such as phishing, malware, and baiting.			[6,20,62,66,67]	
	Training that lacks blended skill development is ineffective in achieving a sustainable goal to mitigate cyber exploitation and ensure personal development for health care professionals.			[7,27,67]	
**Shortage of skilled professionals and limited budget**			15 (20)		
	Another reason for increasing cyber breaches of sensitive health information is the limited budget allocation for cybersecurity.			[1,4,43,47,52,68-70]	
	Health care organizations endure poor security management in containing attacks and cybercrime, and developing new strategies to counteract cyber threats and breaches due to a shortage of skilled professionals and limited budget.			[18,41,63,71]	
	The shortage of cybersecurity experts in health care organizations creates a vacuum for attacks and breaches, while also hindering the development of cybersecurity knowledge among employees.			[43,54,72,73]	
**Security culture**			11 (15)		
	Lack of security culture awareness among health care organization staff, coupled with inadequate training in behavior, interactions, and meaningful work practices within the work environment, constitutes a significant factor that may facilitate improper data handling practices and protection.			[74-76,96,97]	
	Novel viral infections and pandemics requiring rapid technological advancement in health care diagnostics invariably affect behavioral patterns at work and the daily cybersecurity activities of employees.			[77,78]	
	Poor management of organizational culture may affect employees’ cybersecurity behaviors and attitudes toward technology use, thereby increasing the risk of cyberattacks.			[70,78-81]	

**Table 4:** Process factors.

Processes			Studies, n (%)		Reference
**Untimely incident response and recovery plan**			12 (16)		
	Ineffective operational communication systems create poor incident response and preparedness to respond to threats and cyberattacks.			[58,61,82-84]	
	Containing an attack and a breach in a health care system through postincident response takes approximately 100 days or more before a health information system is restored to normal, safe mode.			[85]	
	Cybersecurity strategies in health care systems are often reactive instead of proactive in cyber defense mechanisms, backup, and recovery.			[22,86,87,98]	
	There is limited research on cybersecurity response strategies, which is a great concern.			[87-89]	
**Inadequate policies and procedures**			11 (15)		
	Standard policy protocol for most health care organizations is inadequate to meet best practice measures in cybersecurity.			[42,44,92-94]	
	Some policies and procedures set out by regulatory bodies are cumbersome in laying down information security expectations and are complex to follow. For example, breaches below 500 are neglected and not taken into account.			[61,62]	
	Policies in line with secure behavioral awareness are inadequate for safeguarding health care systems from cyber breaches.			[43,80,90,95]	
**Lack of regular audits and assessments**			10 (13)		
	Most health care organizations do not perform regular or consistent security audits and risk assessments as required by regulations and best practices to visualize security risk levels.			[4,6,22,45]	
	Most health care organizations do not categorize their risks into external and internal risks or have an effective risk plan in place.			[4,52,84]	
	Conducting an assessment and audit of a complex sociotechnical system in cybersecurity fails to factor in technology, organizational environment, and humans as a whole.			[17,22,25,26,84,91]	

### Technology Factors

#### Integration of New Technology

Smart health care systems have successfully procured and integrated medical cyber-physical systems technologies with the Internet of Things to facilitate operations using virtual networks, applications, and devices, as well as to monitor diagnoses, manage treatment, and manage administrative processes in the delivery of health care services [11]. This new technology integration has helped to streamline health care for effective service delivery. The integration of these digital technologies has evolved as they create complex interconnected ecosystems, making it challenging to implement and maintain robust security measures across all components [16,27,41,44,45,47].

Inappropriate technology integration increases the vulnerability of health care organizations to cyberattacks and breaches when the complex STSs integration process and standards are not properly followed or managed [9,42,90]. Additionally, it poses a risk when data is exchanged between the cloud and electronic records, or when it travels within the health care delivery ecosystem. Some of the reasons for the risk are unsupported integration, inappropriate standard implementation [43,46], lack of secure development in the ideation stage [107], ineffective communication, and interoperability issues. These issues, in turn, can give cybercriminals unauthorized access to health information or data because of such vulnerabilities in technology [64]. Furthermore, it is necessary for health care system actors to know that the integration of medical devices and interconnectivity does not equate to interoperability; likewise, interoperability does not equate to the security of medical devices and data protection.

#### Complex System Design and Usability

Complex design and usability can lead to security vulnerabilities in health care information systems [9,104] by affecting data processing, confidentiality, availability, integrity, and design limitations. It creates friction for staff, which can lead to unhealthy security practices in monitoring the IoMT devices and compromising patient safety and privacy [44,50,51]. Additionally, complex and poor system design can make it easier for hackers to exploit vulnerabilities in medical devices and systems, resulting in cyber incidents such as phishing attacks or other social engineering tactics to trick users into giving up their login credentials or downloading and executing malicious software [47,85]. This can harm patients in an emergency and slow care delivery, which can be linked to biomedical nonmaleficence principles [108]. In managing complex health IT challenges, adopting a user-centered approach to health care service operations is pivotal for preventing vulnerabilities and cyberattacks in health care systems [12].

Complex designs and user interfaces of health care devices and applications make it difficult to secure the valuable information in health care systems. Poor design and usability can lead to human user errors, such as accidentally exposing sensitive patient information or mistakenly changing critical medical settings or configurations. The emerging usability literature has highlighted these sociotechnical shortcomings, which could lead to threats and medical errors in health care systems [68]. User satisfaction—whether for patients or health care professionals—at every stage of task performance is enhanced by a friendly design process that prioritizes usability [1,28], design, and data processing. This, in turn, facilitates the effective and efficient delivery of health care services.

#### Third-Party Applications and Plugins

The adoption of third-party applications and plugin software in modern-day smart health care systems can be used in many more ways than traditional standalone software in health care delivery. Third-party application software, in the form of software as a service, has evolved to make use of web-based, intelligent chatbots and large language models. The complexity of these technologies makes it difficult to control their service dynamics as they become vulnerable to cyberattacks [42,51,70,109]. In some cases, the vulnerability of cyber-critical systems that expose health information and patient privacy is not only an issue of the medical device, but also a software malfunction that could put organizations at risk and affect the quality of services [58,61,110].

Hackers can embed malicious software, such as ransomware, in application software or operating systems. Such malicious software can execute and replicate viruses in health care systems by acting like a legitimate third-party software program. It can then create a backdoor to gain access to sensitive information and organization files for launching cryptolocker attacks [56,111]. Additionally, cybercriminals use third-party software and application plugins to impersonate health care service providers, all the while having malicious motives as part of organized syndicates illegally collecting health data. Some medical applications hosted on mobile systems are illegitimate third-party apps, which are another source of privacy violations and data leakage [59,71,112,113].

Malware can easily be introduced to the medical network of systems when the IT team of the medical device software application makes an error during the development stage. It is estimated that 90% of incidents or breaches occur through exploiting vulnerabilities in a device system’s software application program [114]. The use of implanted devices always has issues of software malfunction and update-related problems [1]. For instance, a 2013 analysis of mobile medical health fitness apps showed that over 40% of paid medical applications were completely lacking privacy policies, and 40% of the applications stored sensitive patient information, such as financial details, biodata, and addresses [60]. While only 50% of mobile apps encrypt the personal identifying information sent over the internet, 80% of these third-party applications store this personal identifying information on a local device without encryption, which is liable to be accessed [115]. Having control over third-party software applications and systems while also focusing on developing software from the same device manufacturer will help curb the risk of data breaches and protect sensitive health care–related information [42].

Researchers seem to relate cyber issues to medical devices, neglecting the fact that without operating systems and application software, medical devices would not execute other clinical functions and administrative services in delivering health care [57,58,83]. Regularly updating system software is necessary to improve security against new threats and viruses, since over 90% of breaches stem from programmable software applications or boot systems kernel development, which can be used for implanting viruses in computer systems.

#### Limited Monitoring

Limited monitoring of the health care systems’ critical infrastructure increases the risk of delayed detection of threats and vulnerabilities, allowing them to propagate in the system and cause even greater damage [4,42,52]. Perimeter monitoring technology, such as antivirus and firewalls, also called detection technology, has been developed to recognize known variants of viruses and other threats. In the era of fast-paced technology advancement, ransomware coders are also advancing with detection technology by reprogramming malicious code so that it can remain undetected by the monitoring scanner [52]. Despite the advancement of technology, many health care organizations are still using traditional security monitoring procedures to protect sensitive information and health care systems. Continuous monitoring of health care systems in both real-time and offline modes is essential to enable detection and mitigation of threats [4,42,65].

#### Inadequate Access Control Management

New technology in health care systems requires role-based access control management for professionals and organizations in managing sensitive resources and operations. Many health care organizations become victims of health information breaches or cyberattacks due to inadequate access control management across different technology platforms and applications. This creates a weak access point for cybersecurity operational integration, which results in system flaws, compatibility issues, and interoperability challenges that facilitate access for cybercriminals to gain entry into the health care system network. Strong access control policies help foster effective access control and identity management [6,28,84]. Managing employee privileges and training them not to share passkeys can help prevent lapses in access authorization while ensuring role-based access control to strengthen identity and access management in health care systems [71].

Health care organizations must ensure that their network has strong control systems and structures for better identity management to avoid unauthorized access, breaches of sensitive information, and identity theft [52,67,91]. Weak cybersecurity control and identity management could stem from software applications, human factors, and organizational management processes as a result of outdated systems and technology [69,116-118].

### Human Factors

#### Insider Threats

Insider threats have recently been seen as a growing challenge. Research has attributed these specific threats to the emergence of connected health care IT, which is one of the causes of data breaches or leakages of protected health information [42,119]. However, insider threats are linked to the human element of health care IT systems, wherein human error has been seen as one of the major sources of vulnerabilities in the critical cyber infrastructure [19,67,96]. The root causes of insider threats include insecure behavior by employees and organizations’ inadequate investment in employees’ cybersecurity skills for social and technical know-how [80,81,120]. In contrast, during the era of nontechnical application of care delivery, insider threats were less visible to organizations when protected health information was filed through paper-based manual storage systems. The traditional breaches from insider threats were physical breaches, such as the theft of patients’ valuable information, theft of files and computers, or missing paper health care records [9,11,52,63]. The missing data or breach in patient information was known only to the health care organizations, so the collection of new health records from patients would begin without the need to notify patients about General Data Protection Regulation or Health Insurance Portability and Accountability Act violations [95].

Research has also revealed that since the emergence of the interconnectivity of records, the level of insider threats and attacks has increased tremendously, as such interconnectivity provides multiple gateways for access in a remote location and setting [9,16,61]. Furthermore, the level of insider threats in this era of digital health processes will be more accountable with proper cybersecurity systems and monitoring compared to the paper-based process, where the insider goes unnoticed and underreported. Research has also revealed that, between 2019 and 2024, organizations reported that insider threats increased from 66% to 74% [119]. The literature has also revealed that insiders, rather than outsiders, contributed to about 70% of data fraud and breaches in an organization [86]. This is also attributed to a lack of employee cybersecurity ethics, management implementation of data integrity, and privacy of patient records as a culture of ethics in the workplace [108]. Authors have highlighted different issues of insider threats, digging deep into the risks and issues of insider threats and breaches in health care organizations [67].

#### Inefficient Training

Inefficient training of employees can have a significant negative impact on health care systems, most importantly when a health care professional lacks the knowledge and understanding of cybersecurity vulnerabilities and threat patterns of the health care system [1,52]. It is the duty of health care organizations to give proper training and awareness of cyber threats and attacks to their staff [64,65]; otherwise, employees may easily become vulnerable, resulting in data breaches of sensitive health information [70]. It is important to conduct training assessments for employees; otherwise, it will be difficult to ascertain the extent of the training required [62]. Phishing training, including gamification-based methods, is one approach to assessing employee knowledge. Training results can then be used to design a curriculum that is tailored to work processes, ensuring that employees acquire the training needed to enhance IT security awareness and readiness [42,67]. It is important that health care professionals who use critical hospital infrastructure are trained in comprehensive cybersecurity user applications, including sociotechnical techniques for dealing with health care cybersecurity vulnerabilities, threats, and risks [6,27].

#### Shortage of Skilled Professionals and Limited Budget

Cybersecurity breaches in health care increase daily due to a growing shortage of skilled professionals and limited budgets, posing a significant concern [69,70,73]. This concern is critical for health care organizations due to the large amounts of valuable sensitive data stored in the EHR system and cloud. This sensitive data includes medical records, insurance information, and financial data [16].

Many health care institutions lack the cybersecurity expertise required to defend their digital health care systems from cyberattacks [5,9]. However, while the demand for cybersecurity experts in health care is high, the supply is low. As a result, health care organizations may be subjected to complex assaults on critical infrastructure requiring specific knowledge [54,71]. For instance, cybercriminals take advantage of employees’ low skill sets to exploit them [52]. This shortage of skills continues to leave health care organizations challenged in the changing environment of health care systems, which constrains the organizations from detecting and preventing cyberattacks in health care systems [54]. Furthermore, limited investment in cybersecurity systems and technology accelerates vulnerabilities, threats, and attacks in health care organizations [1,47,104] due to obsolete techniques that lag behind digital trust and security protection. In some cases, health care businesses have limited cybersecurity budgets, making it difficult to invest in the required technologies and resources for defending themselves against threat actors and vulnerabilities [4,43,68]. The shortage of skilled professionals and limited budgets can lead to major cybersecurity vulnerabilities in the health care system [52,69].

#### Security Culture

Security culture plays a crucial role in addressing cyber threats in health care organizations. To properly protect information assets, information security behavior is essential [79]. The norms, values, and attitudes of health care professionals contribute to the development and maintenance of a robust security culture in health care organizations that actively support security initiatives [121]. Thus, employees’ behavior with regard to data privacy is important for the effectiveness of cybersecurity in the workplace environment [70]. Insecure behavior has been identified as one of the most significant factors contributing to vulnerabilities in cybersecurity [76]. Its 4 key components are lack of awareness and experience, unauthorized workflows, behavior prioritization, and environmental appropriateness [80,81].

In this digital health care era, the social influence of peers is a critical driver that influences health care professionals’ motives regarding data privacy policy and security. Furthermore, attitude plays a mediating role in employees’ motives regarding compliance with data privacy and policy [97]. Digitalization in health care organizations can be influenced by attitudes toward cybersecurity, subjective norms, and perception of control over security measures [9]. Insecure behaviors and attitudes of employees and patients regarding the use of technology increase vulnerabilities to cyberattacks.

### Process Factors

#### Untimely Incident Response and Recovery Plan

Untimely incident responses and recovery plans in the event of health information breaches and cyberattacks in health care systems undermine public, stakeholder, and patient trust that health care organizations or hospitals can manage their sensitive health information [84,85,111]. A planned or coordinated response and recovery strategy determines the health care systems’ ability to contain breaches or threats [70,88]. Effective response and recovery plans can mitigate the severity of cyberattacks in health care systems, reducing their impact and preventing future occurrences [71,82]. Despite this, many health care organizations ignore incident response and recovery plans as part of their cybersecurity strategy and measures for protecting health care systems [84,122].

The WannaCry cyberattack incident against the UK’s National Health Service metamorphosed to infect larger systems of health care. This was due to the negligence and poor response strategies associated with the attack [87]. Although the National Health Service management was informed of the vulnerability of the Windows operating system, the IT team was slow to respond to updating the legacy system [52,69]. To mitigate both visualized and hidden cyberattacks in health care systems, the cybersecurity IT team must establish an effective response strategy that integrates evolving technological advancements with new approaches to advanced persistent threats [58,116].

In some cases in which health care organizations were attacked with ransomware, the organizations lost all health care data when they refused to pay a ransom to a cybercriminal. This was due to the lack of a contingency plan, backup, and recovery systems [42,61,85]. Health care organizations are expected to have backup and recovery plans that enable failover of health care data in the event of a cyberattack [12,83] to avoid disruption of services [82].

#### Inadequate Policies and Procedures

Many health care organizations still operate under traditional information security policies and procedures despite technological advancements and the increase in health care breaches and cyberattacks. Traditional information security policies and old-order operational procedures have become obsolete as technology has evolved [93]. Security policies and operational procedures form the foundation for health care systems’ defense against cyber threats and vulnerabilities because they dictate how sensitive health information is protected, incidents are handled, and employees are trained on cybersecurity programs to ensure best practices [52,92,95]. Inadequate policies and procedures predispose health care systems to the risk of cyberattacks and threats [121]. Inadequate policies can stem from several factors, such as underestimation of cyber threats, lack of awareness to engage with cybersecurity issues, and underinvestment [85]. For example, Health Insurance Portability and Accountability Act regulations state that cybersecurity breaches affecting fewer than 500 people should not be reported or fined, which can create ambiguity and gaps in enforcement [61,62]. Additionally, this may encourage organizations with fewer than 500 patients to neglect the security and privacy of this group of patients. Such organizations might endure breaches without disclosing them to the necessary data protection and regulatory authority. The 2015 Anthem breach is a case study of one of the largest breaches, in which the personal information of over 78 million individuals was exposed as a result of inadequate encryption, weak access control policies, and human error [70].

As technology develops, some health care organizations fail to implement new policies that align with evolving technology and the compliance standards necessary to protect health care systems and ensure resilience in managing health information and the entire ecosystem [42,44,61,69,104].

#### Lack of Regular Audit and Assessment

Existing research has shown that many health care organizations conduct security audits and assessments once a year. Health care organizations that do not engage in regular and comprehensive cybersecurity audits and risk assessments often fail to identify cyberthreats and vulnerabilities in health care systems [4,91]. Furthermore, in the absence of regular security audits and assessments health care organizations may struggle to detect vulnerabilities, making it easier for cybercriminals to exploit the weaknesses in their systems [45]. For instance, the cause of the SolarWinds supply chain attack, in which the back door was created by a cybercriminal without detection, is a case in which sensitive information was harvested for more than a year before being detected only after the cybercriminals exposed the information in the public domain. A regular audit ensures the proper monitoring and evaluation of employee behaviors and security practices [84]. Additionally, with these measures, health care organizations can easily detect vulnerabilities and risk levels of third-party applications through comprehensive and regular audits of the health care systems [42,45,91].

Health care organizations that do not conduct monthly and quarterly audits and assessments will significantly increase their cybersecurity risk profile, which may lead to the possibility of continual breaches [42,71].

### Taxonomy Factors of Vulnerabilities to Cyberattacks

Table 5 indicates the taxonomy-related factors of vulnerabilities to cyberattacks, unit-related factors of vulnerabilities to cyberattacks, types of cyberattacks, and their effects on health care organizations.

**Table 5:** Taxonomy factors of vulnerabilities to cyberattacks.

Factors of vulnerabilities to cyberattacks			Unit factors of vulnerabilities to cyberattacks		Types of cyberattacks		Effect on the health care organization		Reference
**Technology**									
	New technology integration	EHRs^a^, medical and network devices, and software		Ransomware, cryptojacking, and DOS^b^		Health information breaches, legal fines from regulators, operational disruptions, data loss, and reputation damage		[1,16,41,45,52,61,70]	
	Complex system design and usability	EHRs, medical and network devices, and software		Ransomware and DOS		Operation disruptions, cyber breaches, loss of trust, legal fines from regulators, financial loss, and reputation damage		[27,43,51,61]	
	Third-party application and plugin	EHRs, medical and network devices, and software		Phishing, DOS, and ransomware		Cyber breaches, health care security weakness, operational disruption, compromised safety, and data loss		[42,61,66,109]	
	Limited monitoring	EHRs, medical and network devices, and applications		DOS, worm infection, ransomware, and data exfiltration		Patient safety risk, service disruption, data breaches, data loss, compromise of Confidentiality, Integrity, and Availability, and operational handicap		[52]	
	Inadequate access control management	EHRs, medical and network devices, and applications		Ransomware, DOS, privilege escalation attack, and phishing		Patient safety risk, data breaches, identity theft, manipulation of data, and possible ransom payments		[16,42,62,66,92]	
**Humans**									
	Insider threats	Health care professionals and EHRs		Identity theft, espionage, and sabotage		Service disruption, loss of trust, sale of data, sensitive data breaches, and data loss		[16,25,42,52,61,70]	
	Inefficient training	Health care professionals		Phishing, worm infection, and ransomware		Financial loss, fine imposition, huge cost implication, data loss, incorrect diagnosis, and error treatment		[27,43,46,70]	
	Shortage of skilled professionals and limited budget	Health care professionals		Ransomware, viruses, phishing, and DOS		Patient safety risk, decreased secure care quality, inadequate compliance, insecure health care services, budget reallocation, and data breaches		[27,46,52,61,63,72]	
	Security culture	Health care professionals		Ransomware, virus, phishing, DOS, DDOS^c^		Insecure behavior, reputation damage, loss of trust, identity theft, security negligence, data breaches, and poor service delivery		[70,77,80,81,97]	
**Processes**									
	Poor incident response and recovery plan	Health information, medical devices, applications, health care professionals, and patients		Ransomware, crypto jacking, DNS^d^ spoofing, and DOS		Health information breaches, identity theft, legal suits, health care service disruption, ransom payments, loss of data, hard-to-recover data, and financial loss		[43,82,85,98,123]	
	Inadequate policy and procedure	Health information, medical devices, applications, health care professionals, and patients		Ransomware, worm infection, phishing, and DOS		Service disruption, possible patient harm, compromised sensitive data, regulatory fines, violation of privacy, financial loss, poor security strategies, and data breaches		[43,61,62,92,95]	
	Lack of regular audit and assessment	Health information, medical devices, applications, health care professionals, and patients		Man-in-the-middle attack, crypto jacking, and worm infection		Reputation damage, possible patient harm, service disruption, privacy violation, unauthorized access freedom, breaches of sensitive information, and data loss		[42,52]	

^a^EHR: electronic health record.

^b^DOS: denial of service.

^c^DDOS: distributed denial of service.

^d^DNS: domain name system.

## Discussion

### Summary of the Findings

This study examined the dynamics of the factors of vulnerability to cyberattacks in the 3 core areas of the STSs theory of technology, humans, and processes in health care systems through a scoping review of 76 papers.

This study found that the integration of new technology can be challenging in protecting health care systems from cyberattacks in the absence of an appropriate intervention. The findings also showed that complexities in system design present adaptability challenges for health care professionals; thus, cyberspace is prone to a high-risk incidence of threats. Furthermore, third-party software limits security in smart health care, which has various impacts on health organizations.

The findings revealed that internal threats existing in health care systems are not linked only to health care professionals but also to IT teams. Additionally, inefficient cybersecurity training exposes health care organizations to vulnerabilities and cyberattacks. The findings also showed that inadequate investment in human capital and limited finances contribute to poor cybersecurity management. This study further found that the decline in security culture is based on cultural deviation and radical technological change in health care organizations, which is deeply rooted in the behaviors and attitudes of employees.

The present study found that most health care organizations are unprepared and do not have a proactive incident response and recovery plan in place in the event of a cyberattack [85]. The communication gap, untimely postincident response, cybersecurity strategies, and limited research on cybersecurity responses contribute immensely to cyber threats and cyberattacks. Furthermore, limitations in continuous monitoring include inadequate capabilities, inconsistent monitoring, and complex monitoring processes that increase cyber insecurity in health care organizations. The findings also indicate that cybersecurity policies and procedures can be complex and inadequate in shaping the security of health care cyberspace. Additionally, cybersecurity auditing and assessment can be inconsistent, fail to classify risks as internal or external, and include nonholistic perspectives of the STS. The study further found that weak access control management and breakdowns facilitate the exploitation of sensitive data in health care systems.

The findings showed that despite the similar unit factors of vulnerabilities to cyberattacks for the subfactor of technology and the occurrence of various types of cyberattacks, the effect on health care organizations remains the same. Additionally, despite the similar unit factors of vulnerabilities to cyberattacks for the subfactor of humans, the types of cyberattacks that occurred differed to some extent; however, the effect on health care organizations was somewhat varied. Furthermore, despite the similar unit factors of vulnerabilities to cyberattacks for the subfactors of processes, the types of cyberattacks that occurred were similar to a great extent; consequently, the effects on health care organizations were also similar to a great extent. In general, this study found that processes involve both technology and humans relative to the unit factors of vulnerabilities to cyberattacks. This confirms the sociotechnical interplay among the factors of vulnerabilities in health care systems [17].

### Sociotechnical Cybersecurity Framework

#### Overview

The three core constructs of STSs that can protect health care systems from vulnerabilities to cyberattacks and breaches are technology, humans, and processes [23,25]. In the context of this study, the three constructs of STSs are referred to as the factors of vulnerabilities, which are the areas in which vulnerabilities occur.

**Figure 3:**
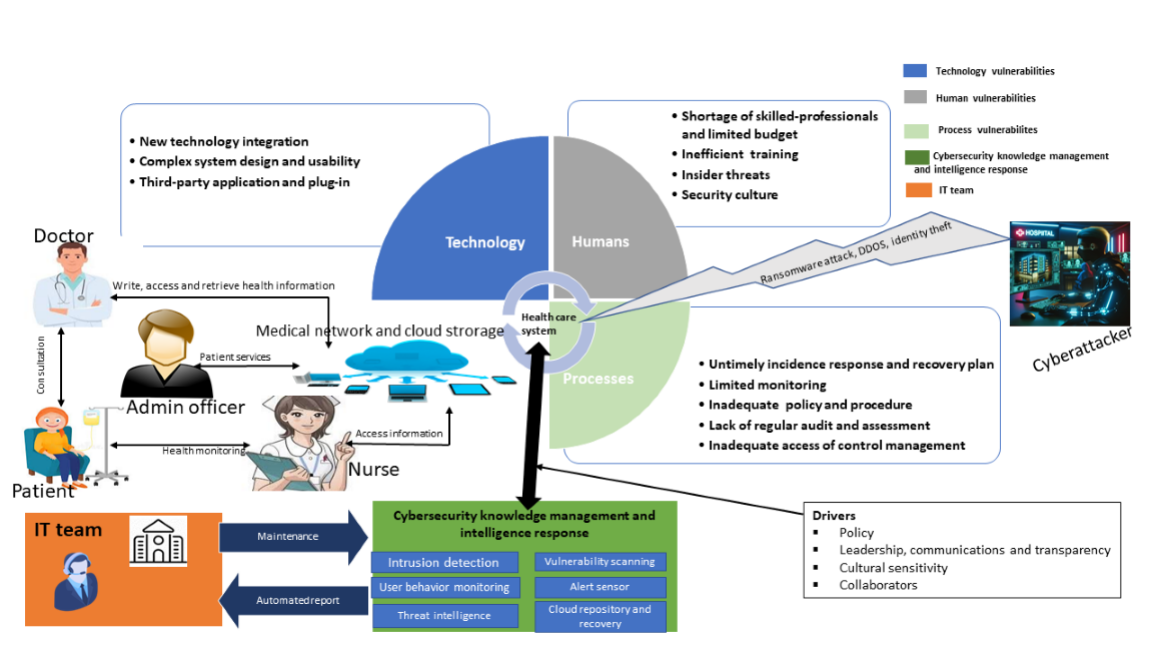
Conceptual sociotechnical cybersecurity framework. DDOS: distributed denial of service; IT: information technology.

This study proposed a conceptual sociotechnical cybersecurity framework for health care systems that entails the factors of vulnerabilities, IT team, cyberattackers, and cybersecurity knowledge management and intelligence response (CKMIR). The framework incorporates features such as intrusion detection and response, user behavior monitoring, threat intelligence, vulnerability scanning, alert sensors, cloud-based repositories, and recovery mechanisms as a comprehensive approach in responding to the vulnerabilities, cyberattacks, and threats in health care systems; this framework is presented in Figure 3.

The components of the sociotechnical cybersecurity framework are explained in the following sections.

#### Factors of Vulnerabilities

The factors of vulnerabilities involve humans, technology, and processes, which are interwoven in the sociotechnical cybersecurity framework [5,54,87].

#### IT Team

The IT team is one of the human elements in the loop that provides technical support, maintenance, and remediation for the health care system. The IT team includes software engineers, system developers, cybersecurity experts, compliance officers, IT support staff, and network engineers. They are responsible for the day-to-day health of IT operations to ensure smooth and secure health care service delivery.

#### Health Care Professionals

Health care professionals include doctors, nurses, administrative staff, etc. The doctors consult with the patients online and onsite, access their medical history from the cloud through the EHR system, and prescribe medication, while the nurses monitor patients’ health, provide care, and access patients’ medical information through the medical network. Health care administrative staff are responsible for administrative and clinical tasks, such as scheduling staff and appointments for patients to ensure the practice runs smoothly.

#### Cyberattackers

The cyberattacker is a cybercriminal who exploits the health care system using sophisticated techniques to launch attacks on health care–critical infrastructure. They launch attacks through denial of service, ransomware, and identity theft of patient health information. The stolen information is sold on the dark web for financial gain.

#### About CKMIR

The CKMIR intrusion detection feature systematically analyzes network traffic, human behavior, technology, and processes in real time to optimally detect and isolate known and unknown cyber threats and attacks in health care systems to enable remediation.

The CKMIR user behavior monitoring feature identifies and analyzes the patterns of human behavior and interactions within health care systems, such as login times, access patterns, file transfers, and application usage, as well as internal and external threats, to determine unauthorized access and compromised accounts.

The CKMIR threat intelligence feature collects, analyzes, and interprets raw data on the intent, opportunity, and capability of malicious actors and shares structured information with the IT team through actionable intelligence.

The CKMIR vulnerability scanning feature scans, detects, identifies, and classifies technology, human, and process factors of vulnerabilities in health care systems and provides countermeasures for cyber threats.

The CKMIR alert sensor senses isolated cyber threats and attacks and sends alerts to the IT team in real time.

The CKMIR cloud repository and recovery feature store and back up encrypted data, critical system files, and security event records to recover data in the event of a cyberattack.

#### Drivers

The drivers are the factors that determine the transition of cybersecurity in health care organizations. They play critical roles in shaping sustainable cybersecurity in health care systems. These drivers include policy, leadership, communications and transparency, cultural sensitivity, and collaborators.

In this conceptual framework, CKMIR plays a significant role in automated defense regarding vulnerabilities and intelligent response in the event of a cyber threat or attack.

The framework provides a contemporary foundation and pathway for identifying and preventing vulnerabilities and responding to cyberattacks and threats in health care systems. This conceptual framework is important for identifying, capturing, organizing, storing, and sharing real-time data and actionable intelligence and preventing vulnerabilities to cyberattacks in health care systems. The conceptual framework functions holistically from a sociotechnical perspective of cybersecurity in health care systems. The proposed framework plays a critical role in system interplay for detecting, classifying, and preventing vulnerabilities and providing real-time incident response and automated report generation to ensure that the IT team is informed of the current security status, ongoing incidents, and actions taken.

**Figure 4:**
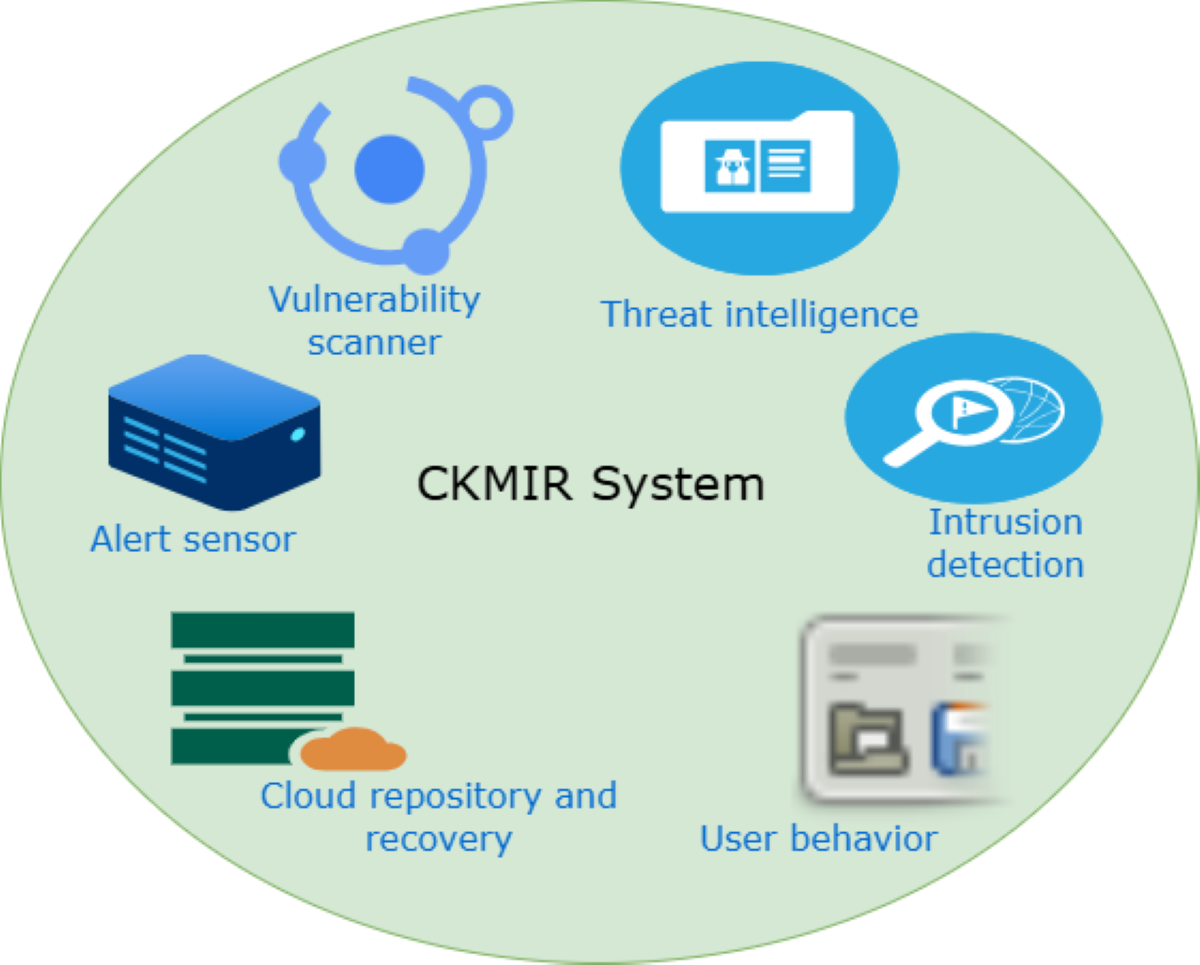
Visual model: proposed CKMIR system. CKMIR: cybersecurity knowledge management and intelligence response.

In Figure 3, an up-down bidirectional arrow indicates the relationship between CKMIR and health care systems. This up-down bidirectional relationship shows that CKMIR prevents vulnerabilities, provides real-time incident response, stores data, and remediates it in the event of threat intrusion and cyberattack, while the health care systems transmit data to CKMIR. Furthermore, opposing 2-way arrows show a relationship between CKMIR and the IT team. This 2-way relationship indicates that CKMIR transmits automated reports while the IT team accesses CKMIR to perform maintenance, remediation, and decision-making. In essence, this framework offers a comprehensive and well-defined approach to the sociotechnical underpinning and joint optimization of cybersecurity’s progress in achieving sustainable health care systems. The visual model of the proposed CKMIR system is shown in Figure 4.

### Practical Implementation Steps for the Conceptual Framework

The practical implementation steps for the validation of the proposed conceptual sociotechnical cybersecurity framework are shown in Multimedia Appendix 6. The implementation steps involve the classification of the vulnerability’s areas of occurrence (technology, humans, and processes), defining goals, mapping stakeholders, orientation, risk assessment, validation, and feedback. The guide indicates an interplay within the vulnerability’s areas of occurrence (technology, humans, and processes). It also shows that there is a joint optimization between the vulnerabilities’ areas of occurrence and the CKMIR system to identify and prevent vulnerabilities and respond to cyberattacks. The implementation of the proposed sociotechnical cybersecurity framework for health care systems (hospitals) in a real-world scenario is aimed at achieving optimal cybersecurity resilience.

### Linking the CKMIR System to the NIST Model

**Figure 5:**
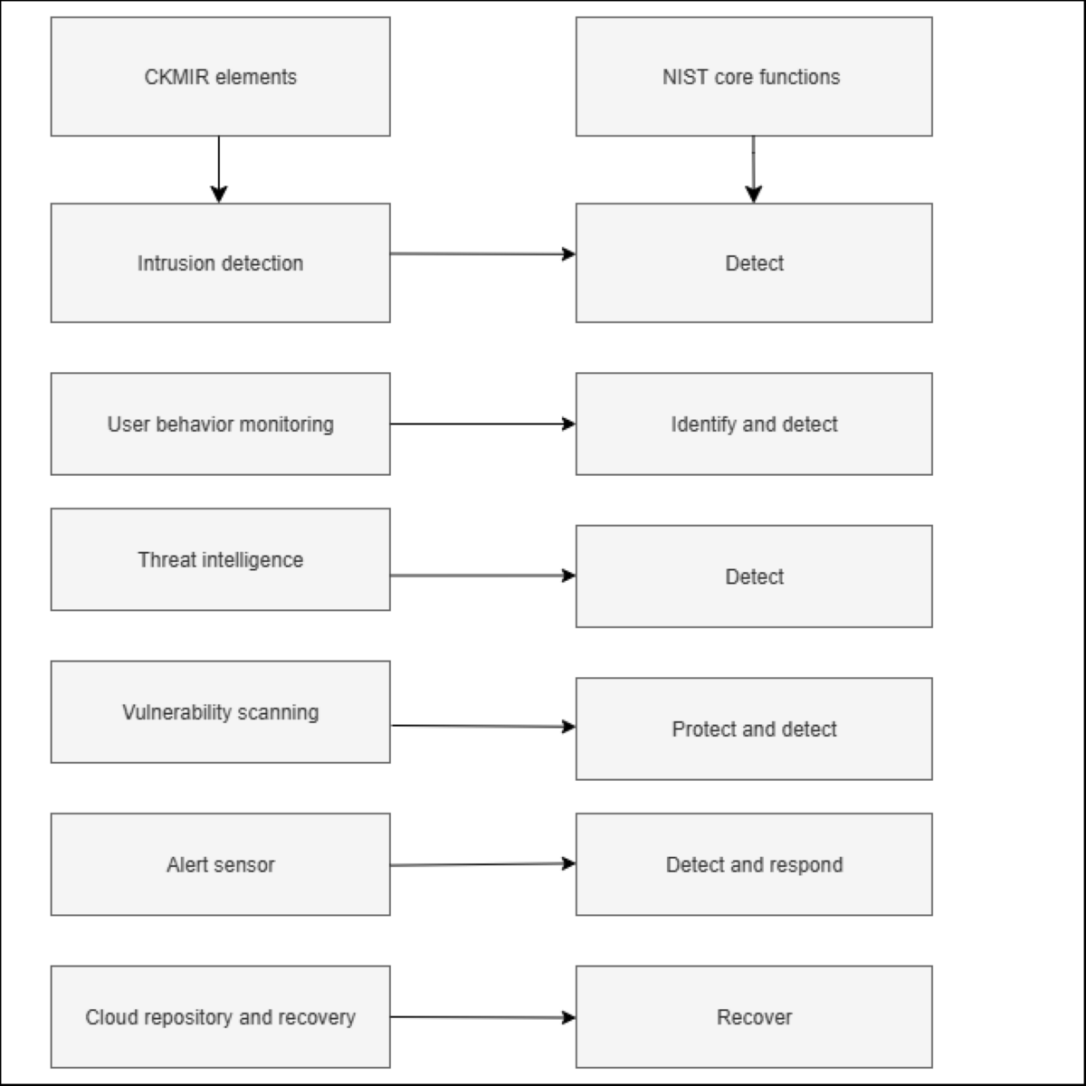
CKMIR element alignment with the NIST model. CKMIR: cybersecurity knowledge management and intelligence response; NIST: National Institute of Standards and Technology.

The CKMIR elements align with the core functions of the NIST model in Figure 5. The core functions of the NIST model involve identifying, protecting, detecting, responding, and recovering [124]. The CKMIR elements involve intrusion detection, vulnerability scanning, user behavior monitoring, alert sensors, threat intelligence, and cloud repository and recovery.

The unique value proposition of the CKMIR model is the configuration, dynamic integration, and its mode of operation, such as real-time incident response optimization. Specifically, its unique value proposition is the provision of threat intelligence, human behavior analytics, and cross-component integration in the health care system. The CKMIR model applies to the health care system in its capacity to solve complex health care problems in the vulnerable areas of occurrence emanating from IoMT devices, cloud, EHRs, health care professionals, and patients. The model-specific sociotechnical contributions encompass the optimal identification and mitigation of vulnerabilities arising from technology, humans, and processes.

### Compliance Standards for Applying the Proposed Framework

Compliance standards are necessary for the application of the conceptual sociotechnical cybersecurity framework to guide health care organizations in their cybersecurity practices. It will also facilitate the process of cybersecurity risk assessment for health care professionals. The compliance standard is detailed in Multimedia Appendix 7 [12,22,25,42,44,45,52,53,61, 63,70-72,75,76,80,81,87,95,99,125].

### Practical Implications

Considering the increase in cyberattacks, breaches, and overdependence on modern technology for health care diagnosis and treatment, it is important for health care organizations and stakeholders to examine how technology can be implemented. In particular, policies should mandate secure development for technology integration and third-party applications through adoption and control measures within health care system audit assessments and compliance procurement plans. Health care organizations should leverage the strength of cybersecurity through the implementation of risk assessment and incident response plans that complement current and emerging threats and cyberattacks. Health care organizations should adopt compliance standards for applying the sociotechnical framework as a guide to maintaining cybersecurity hygiene in health care systems (Multimedia Appendix 7). Health care institutions should ensure that the implementation of a medical device security lifecycle is integrated into Confidentiality, Integrity, and Availability practices as quality control measures [21]. Health care organizations should implement network segregation of sensitive areas for greater protection, easy usability, and secure workarounds. Additionally, regular network assessment is required to monitor traffic and network behavior, and to trigger alerts regarding abnormalities [50]. The design of network systems should be simplified and while training professionals to develop secure health care systems. Further, health care management should recruit more skilled professionals, offer training to employees, and increase budgeting for cybersecurity to ensure the delivery of uninterrupted health care services. Health care organizations must implement strong access control systems and policies that ensure the use of strong password systems, multifactor authentication, and strong privileges that grant access to health care critical infrastructure only to authorized employees.

The adoption of the sociotechnical cybersecurity framework by health care organizations will accelerate and optimize cybersecurity progression and support IT teams and operational processes in sustaining the health care cyber space.

### Comparison With the Previous Literature

The findings of the scoping review are in line with the existing evidence that obsolete infrastructure, limited budget, complex policies and procedures, ineffective training, and a shortage of cybersecurity experts are barriers to cybersecurity in health care systems [1,68,72]. Additionally, Al-Qarni [92] affirms our findings that health care organizations must have an evolving policy that aligns with emerging technological trends and cyber threats, along with a continuous upgrade and backup plan.

Various schools of thought advocate addressing cybersecurity vulnerabilities in health care systems through a sociotechnical approach, rather than relying solely on technical or social perspectives. Invariably, studies support holistic and joint optimization approaches [11,17,126,127].

The concept of applying a sociotechnical perspective to cybersecurity in the health care domain has received little attention over the years, and the notion of a sociotechnical perspective on cybersecurity in health care is still evolving. Nevertheless, for cybersecurity in health care, a myriad of perspectives, such as a social perspective [24], a cybersecurity perspective [10,20], a sociotechnical perspective [12], the NIST perspective [45], an organizational perspective [104], and a knowledge management perspective [5] have been applied. In this study, cybersecurity challenges and issues were addressed in health care organizations from an outstanding approach of the sociotechnical viewpoint by developing the sociotechnical cybersecurity framework; this is a novel instance of the theoretical contributions (Figure 3).

In the quest for solutions, scholars have developed various frameworks that contribute to the theory of cybersecurity in health care. Rehman et al [55] proposed a framework for a secure health monitoring system in health care 5.0 and used blockchain technology and an intrusion detection system to detect any malicious activity in health care networks. Wazid et al [53] proposed a framework for generalized secure healthcare 5.0 to provide solutions for the challenges in health care systems. Furthermore, Jalali et al [88] proposed the Eight Aggregated Response Strategies (EARSs) framework for cybersecurity incidents. In this context, the CKMIR model differs from the secure health monitoring model [55] in the configuration of its elements. Further, the CKMIR model differs from the secure healthcare 5.0 model [53] in its capability to respond to numerous simultaneous cyberattacks. Additionally, our proposed model optimized cybersecurity response capabilities compared to the EARS model [88]. The incident reporting and vulnerability analysis are automated and embedded within our model, unlike in the EARS model. Generally, the CKMIR model differs from existing models in its components’ compatibility, design, and joint optimization of the technology, humans, and processes in preventing vulnerabilities and responding to cyberattacks.

This study contributes to existing cybersecurity theory in several ways, taking an entirely different approach. One way is through the thematic classification of technology, human, and process-related factors of vulnerabilities to cyberattacks in health care systems in their descriptive format (Tables 2-4). It highlights the 3 constructs of sociotechnical-related factors of vulnerabilities to cyberattacks relative to their subfactors in health care systems. The second contribution is an in-depth analytical synthesis of the taxonomy factors of vulnerabilities to cyberattacks. It highlights such factors relative to their subfactors in health care systems (Table 5). The main contribution is the development of the conceptual sociotechnical cybersecurity framework for health care systems (Figure 3). The framework identifies and prevents vulnerabilities and responds to threats and cyberattacks. The proposed framework provides the foundation for understanding the connection and integration of the factors of vulnerabilities (technology, humans, and processes) to cyberattacks and threats from a sociotechnical perspective in health care systems. It presents a comprehensive approach that is important for fostering and supporting the current understanding of cybersecurity from a sociotechnical lens in health care systems.

### Limitations

This study included only papers published in English. Gray literature was not examined. Reports, research-in-progress papers, editorial papers, and inaccessible papers were also excluded. Furthermore, papers outside the study’s context were excluded. Cybersecurity in health care papers from a sociotechnical perspective were rarely available.

### Conclusions

The sociotechnical perspective of cybersecurity is a critical prerequisite and foundation for resolving vulnerabilities and preventing cyberattacks, breaches, and threats in a complex health care system. This study used a scoping review to examine the dynamics of the factors of vulnerabilities to cyberattacks and develop the sociotechnical cybersecurity framework for preventing vulnerabilities and responding to threats and cyberattacks in health care systems. Furthermore, this study also presents the compliance standards for the application of the conceptual framework to guide health care organizations’ cybersecurity practices. This study examined the landscape of cybersecurity vulnerabilities and confirmed that an interplay exists among the 3 sociotechnical themes of technology, humans, and processes.

Despite the growing benefits of technology, this study observed that the increasing number of breaches and cyberattacks is linked to the unpreparedness of health care organizations, a lack of compliance, communication issues, irregular adverse assessments, and a lack of timely response to cybersecurity incidents and proper monitoring. It should be noted that online and offline backup and recovery plans are important for mitigating incidents. Health care organizations that embed a culture of inclusiveness and training with the necessary skills can eliminate insider threats and cyberattacks in health care systems. To address the vulnerabilities related to complexities in system design, health care organizations must ensure that priority is given to cybersecurity and user-centered designs for processes and the technological integration, application, and implementation of critical health care infrastructure as a sociotechnical approach [27,54]. This includes implementing security design and multifactor authentication instructions, secure text display, cryptographic instructions, tokenization, and alert triggers to providers and legitimate users to control system security operations. This implementation can affect usability and complex design from the patients’ and providers’ points of view to track intrusions, detect abnormalities, and prevent unlawful access to health information.

The proposed conceptual sociotechnical cybersecurity framework provides a comprehensive and explicit overview of the sociotechnical foundations of vulnerabilities (technology, human factors, and processes) in health care systems.

In spite of the existing generic cybersecurity frameworks from a sociotechnical perspective to tackle issues of vulnerabilities and cyberattacks in organizations, the framework is important for its suitability, applicability, and customization to a dynamic and complex health care system.

In addition to further research to empirically validate the proposed framework for accuracy, feasibility, and effectiveness in health care organizations, there is also a need to investigate the adoption of blockchain technology for accelerating incident response processes in health care systems.

## Appendix

Multimedia Appendix 1 PRISMA-ScR checklist.

Multimedia Appendix 2 Detailed search strategy.

Multimedia Appendix 3 Characteristics of the included studies.

Multimedia Appendix 4 Critical appraisal.

Multimedia Appendix 5 Quality appraisal grouped by study method.

Multimedia Appendix 6 Practical implementation steps for the conceptual framework.

Multimedia Appendix 7 The compliance standards to guide the conceptual framework.

## Abbreviations

CKMIR: cybersecurity knowledge management and intelligence response
EARS: Eight Aggregated Response Strategy
EHR: electronic health record
IoMT: Internet of Medical Things
JBI: Joanna Briggs Institute
NIST: National Institute of Standards and Technology
PRISMA-ScR: Preferred Reporting Items for Systematic Reviews and Meta-Analyses extension for Scoping Reviews
RQ: research question
STAMP: systems theoretic accident model and processes
STS: sociotechnical system
