# Supplementary material for: Sociotechnical Cybersecurity Framework for Securing Health Care From Vulnerabilities and Cyberattacks: Scoping Review
Source: J Med Internet Res. 2025 Oct 15;27:e75584. doi: 10.2196/75584 (PMC12572753; doi:10.2196/75584)
Supplement: Multimedia Appendix 2 [file jmir_v27i1e75584_app2.docx]

**PubMed (Medline)**

( "Computer Security"[Mesh]

OR Cyberattack*[tw]

OR Cybercrime*[tw]

OR "Cyber Crime"[tw]

OR Cyberthreat*[tw]

OR "Cyber Threat"[tw]

OR "Cyber Crises"[tw]

OR "Cyber Risk"[tw]

OR "Cyber Incident"[tw]

OR Cyber Operation[tw]

OR Cyberspace[tw]

OR "Cyber Infrastructure"[tw]

OR "Data Breach"[tw]

OR "Data Security"[tw]

OR "Firewall"[tw]

OR "Information Security"[tw]

OR "Information Technology Security"[tw]

OR "Information Systems Security"[tw]

OR "Security Incident"[tw]

OR "Network Security"[tw]

OR Ransomware[tw]

OR Malware[tw]

OR Phishing[tw]

)

AND

( "Health Care Facilities, Workforce, and Services"[Mesh] OR "Delivery of Health Care, Integrated"[Mesh] OR "Health Care"[tw]

OR "Health Information"[tw]

OR "Health Information Management"[tw]

OR "Healthcare Systems"[tw]

OR "Health Systems"[tw]

OR "Health System Infrastructure"[tw]

OR "Medical Devices"[tw]

OR Medical Technolog*[tw]

OR Health Technolog*[tw]

OR Health Care Technolog*[tw] )

WEB OF Science

(TS=("Computer Security" OR Cyberattack* OR Cybercrime* OR "Cyber Crime" OR Cyberthreat* OR "Cyber Threat" OR "Cyber Crises" OR "Cyber Risk" OR "Cyber Incident" OR "Cyber Operation" OR Cyberspace OR "Cyber Infrastructure" OR "Data Breach" OR "Data Security" OR Firewall OR "Information Security" OR "Information Technology Security" OR "Information Systems Security" OR "Security Incident" OR "Network Security" OR Ransomware OR Malware OR Phishing)

AND

TS=( "Health Care Facilities" OR "Delivery of Health Care, Integrated" OR "Health Care" OR "Health Information" OR "Health Information Management" OR "Healthcare Systems" OR "Health Systems" OR "Health System Infrastructure" OR "Medical Devices" OR Medical Technolog* OR Health Technolog* OR "Health Care Technolog*")) AND (DT==("REVIEW") AND LA==("ENGLISH") AND DT==("REVIEW") AND PY==("2012" OR "2013" OR "2014" OR "2015" OR "2016" OR "2017" OR "2018" OR "2019" OR "2020" OR "2021" OR "2022" OR "2023" OR "2024") AND LA==("ENGLISH") AND DT==("REVIEW"))

**SCOPUS Database**

TITLE-ABS-KEY ( "Computer Security" OR cyberattack* OR cybercrime* OR "Cyber Crime" OR cyberthreat* OR "Cyber Threat" OR "Cyber Crises" OR "Cyber Risk" OR "Cyber Incident" OR "Cyber Operation" OR cyberspace OR "Cyber Infrastructure" OR "Data Breach" OR "Data Security" OR firewall OR "Information Security" OR "Information Technology Security" OR "Information Systems Security" OR "Security Incident" OR "Network Security" OR ransomware OR malware OR phishing )

AND

TITLE-ABS-KEY ( "Health Care Facilities" OR "Delivery of Health Care, Integrated" OR "Health Care" OR "Health Information" OR "Health Information Management" OR "Healthcare Systems" OR "Health Systems" OR "Health System Infrastructure" OR "Medical Devices" OR medical AND technolog* OR health AND technolog* OR "Health Care Technolog*" ) AND PUBYEAR > 2011 AND PUBYEAR < 2025 AND ( LIMIT-TO ( LANGUAGE , "English" ) ) AND ( EXCLUDE ( SRCTYPE , "b" ) OR EXCLUDE ( SRCTYPE , "k" ) ) AND ( LIMIT-TO ( DOCTYPE , "re" ) ) AND ( LIMIT-TO ( PUBSTAGE , "final" ) )
