# Supplementary material for: Sociotechnical Cybersecurity Framework for Securing Health Care From Vulnerabilities and Cyberattacks: Scoping Review
Source: J Med Internet Res. 2025 Oct 15;27:e75584. doi: 10.2196/75584 (PMC12572753; doi:10.2196/75584)
Supplement: Multimedia Appendix 3 [file jmir_v27i1e75584_app3.docx]

Alhassani **Data Charting**

| Author | Year of Publication | Country of Origin | Study Design | Aims | Key findings |
| --- | --- | --- | --- | --- | --- |
| Malatji et al [25] | 2020 | South Africa | Mixed methods - focus group discussions and in-depth personal interviews, and online survey | To validate the management process that identifies and addresses sociotechnical security gaps in existing enterprise systems security frameworks, and how the frameworks’ security controls could be optimized and matured | The study found that a 5-level capability maturity model (CMM) is commonly used by enterprises to carry out maturity assessments of their practices |
| Zimmermann and Renaud [23] | 2019 | South Africa | Qualitative. Problematization approach | To uncover characterization of ‘the problem’ in the cybersecurity arena. | The study found that human actor in cybersecurity is generally considered to be ‘problem’ in the wider sociotechnical cybersecurity system. This problematization revealed that current solutions primarily focus on preventing adverse events by building resistance, implementing new security layers and policies that control humans and constrain their behaviors |
| Malatji et al [17] | 2019 | South Africa | Qualitative Sociotechnical systems cybersecurity framework (STS-CF) development methodology | To develop a sociotechnical systems framework to help identify and appropriately respond to any vulnerabilities that may result from the sociotechnical gaps within the existing information and cybersecurity solutions | The application of the conceptual process model is able successfully categorize the selected information and cybersecurity practices into either social, technical or environmental practices. The analysis revealed that most existing information and cybersecurity frameworks have sociotechnical gap, with an overemphasis on technical aspects and less focus on social dimensions |
| Kandasamy et al [45] | 2022 | India, Indonesia, Saudi Arabia, Hongkong and Singapore | Review | To investigate the recent cyberattacks in Asian health care institutions | Five major types of recent cyberattacks were found to dominate Asian healthcare institutions. A detailed analysis of these attacks, their vulnerabilities, and associated risk are performed |
| Kioskli et al [1] | 2021 | United Kingdom and Greece | Qualitative narrative review | To explore the existing vulnerabilities in the health care critical information infrastructures | There is sparse evidence that by capturing the attackers’ profiles, such as psychological, social and behavioural traits along with the opportunities, capabilities and motives, we can better identify the attackers. For the application of cybersecurity measures to boost cybersecurity practices, the paper revealed that cybersecurity standards are many and sometimes either contradicting and overlapping |
| Dias et al [4] | 2021 | Brazil | Systematic literature review and Bardin's three classic phases of content analysis: pre-analysis, exploration of the material, and treatment of results and interpretations. | to investigate risk management focusing on identifying requirements and best practices for healthcare data security systems. | contributes to defining a set of minimum requirements and best practices that can be adopted to manage data security risks in the healthcare sector and medical devices. |
| Kaberuka and Johnson [6] | 2023 |  | adopts a comparative case study design, applying the System-Theoretic Accident Modeling Process (STAMP). It integrates the NIST control taxonomy. | The main goal of the paper is to highlight how cybersecurity in healthcare is not merely a technical issue but a complex socio-technical problem involving organizational, operational, and governance vulnerabilities. It aims to critique existing risk analysis methods and demonstrate how STAMP augmented by NIST taxonomies can more effectively identify and address systemic cybersecurity risks. | The findings reveal that while the STAMP framework is useful for high-level control analysis, it can be difficult to apply without more detailed support. |
| Khando et al [96] | 2021 | Sweden | Literature review and PDCA and SETA program | The study aims to provide a systematic review of the literature on Information Security Awareness (ISA) and present a state-of-the-art collection of ISA methods and factors for enhancing employees' ISA within both private and public sector organizations. | This study provides a systematic review of literature on Information Security Awareness (ISA) and presents key findings on methods and factors for enhancing employees' ISA within both private and public sector organizations. The authors noted that Management support, education and training, culture, and Information Security Policy (ISP) provision are common factors for enhancing ISA in both private and public sectors. |
| Sittig and Singh [12] | 2016 | United States | Qualitative using a socio-technical framework | The study aims to present a socio-technical approach for healthcare organizations to prevent, mitigate, and recover from ransomware attacks, especially in systems involving electronic health records (EHRs). | The study emphasizes a four-step socio-technical strategy to combat ransomware in healthcare: secure technical infrastructure, train users through simulations, continuously monitor systems for threats, and establish rapid recovery protocols. It highlights that both IT staff and end-users must share responsibility, and recommends following NIST guidelines to enhance electronic health record (EHR) security and resilience. |
| Coventry and Branley [16] | 2018 | United Kingdom | narrative literature review based on a structured search of PubMed articles | The study aims to explore why healthcare is vulnerable to cyberattacks, why it is a target, what current threats and consequences exist, what legislative frameworks are in place, and how the healthcare sector can improve its cybersecurity posture. | The study shows that healthcare is especially vulnerable due to outdated systems, poor cybersecurity funding, interconnected devices, and lack of staff training. It is targeted for its rich, valuable data, with motivations ranging from financial gain to cyberwarfare. The consequences include major breaches, ransomware attacks, operational shutdowns, and loss of patient trust. Legislation like HIPAA and GDPR are helping shape accountability, but implementation gaps remain. |
| Argaw et al [18] | 2019 | Switzerland | scoping review following the Arksey & O’Malley framework, combined with PRISMA guidelines. | The review aimed to map the scientific literature on hospital cyberattacks, categorize domains of research, and extract cybersecurity recommendations relevant to hospitals and healthcare infrastructure, providing foundational insights for healthcare stakeholders and policy forums. | trends in cybersecurity, connected devices, hospital information systems, awareness, security methodology, and attack types (e.g., ransomware). The field is emphasized improved training, secure infrastructure, periodic audits, collaboration with cybersecurity experts, and device-level safeguards, yet highlighted a persistent gap in standardized cure infrastructure, periodic audits, collaboration with cybersecurity experts, and device-level safeguards, yet highlighted a persistent gap in standardized best practices across hospitals. |
| Garcia-Perez et al [20] | 2023 | United Kingdom | Quantitative empirical study with PLS-SEM (Partial Least Squares Structural Equation Modeling) | The study aims to explore how healthcare organizations can build digital resilience during digital transformation by focusing on three core constructs: cybersecurity knowledge and resources, awareness of digital risks, and partnerships/supply chain interdependence. | The study finds that cybersecurity knowledge, uncertainty awareness, and interdependence are critical enablers of digital resilience in healthcare. Organizations with deficiencies in these areas are more vulnerable during digital transformation. The findings highlight the need for strategic governance, supply chain collaboration, and executive-level cybersecurity preparedness. |
| Szczepaniuk and Szczepaniuk [21] | 2023 | Poland | Mix method.  Technical framework and empirical evaluation using software engineering methods | The paper aims to design and test a secure, interoperable architecture for smart healthcare systems by integrating blockchain and cryptographic smart contracts to enhance EHR processing. Key goals include creating a structured EHR format, securing stakeholder interactions, and assessing the framework’s cybersecurity against known threats. | Empirical tests showed high stability and reliable performance, while cybersecurity analysis identified threats like Eclipse and Sybil attacks, recommending strategies using ENISA and NIST guidelines. The study confirms that cryptographic smart contracts and structured EHR models can significantly improve the cybersecurity and resilience of smart healthcare systems. |
| Vukotich G. [22] | 2023 | United States | Zero Trust security framework | The paper aims to address weaknesses in healthcare cybersecurity and promote Zero Trust Architecture as a strategic shift. It seeks to educate leaders on shifting from perimeter-based security to user, data, and system-focused protection across healthcare ecosystems. | The study highlights the rising cost and complexity of cyberattacks in healthcare, emphasizing that outdated perimeter defenses are insufficient. It advocates for a Zero Trust model centered on continuous verification across seven pillars: user, device, application/workload, data, network, automation, and analytics. The author underscores that cyber readiness must evolve with threats, urging proactive audits, system-wide monitoring, and culture change to protect healthcare infrastructure. |
| Svandova and Smutny [26] | 2024 | Czech Republic | scoping review following PRISMA ScR guidelines. | The study aims to map existing IoMT security frameworks, evaluate their focus on technological and organizational measures, and identify research gaps. Two core research questions guided the study: (1) the extent of technological risk assessment and management frameworks for IoMT, and (2) the presence of frameworks that assess information system security in hospitals including organizational aspects. | The study review found most IoMT security frameworks emphasize technical solutions, especially using machine learning for intrusion detection, malware prevention, and anomaly detection. However, none of the frameworks included organizational risk assessments, despite recognizing their importance. |
| Alhammad et al [44] | 2022 | Malaysia | systematic literature review based on Tranfield’s method | The study aims to identify and classify cyber threats to MDI-EMR systems, understand vulnerabilities from technical, organizational, and human perspectives, and assess existing mitigation strategies to enhance security, privacy, and patient safety in interconnected health systems. | The paper finds that the integration of medical devices with EMRs introduces complex vulnerabilities, with threats including phishing, ransomware, SQL injection, spoofing, and denial-of-service attacks. Hospitals face increasing risks, exacerbated by weak organizational safeguards and a lack of cybersecurity awareness. The authors recommend layered controls technical, physical, and administrative and promote frameworks like Microsoft’s STRIDE model to assess and mitigate cyber risks. |
| Giansanti and Monoscalco [99] | 2021 | Italy | Qualitative interview using Questionnaire instrument | The study investigates cybersecurity in cardiology, which is considered a strategic field in healthcare due to its high technological content in both medical devices and care systems | The study revealed several key findings regarding cybersecurity perception among cardiologists:  Safety self-perception: Cardiologists rated the safety of their own environment at 3.58 out of 5 and also the authors also find that Desire for training: There was a strong desire to invest in cybersecurity training, scoring 4.01 out of 5, inadequate cybersecurity initiative, lack of training was also noted among cardiologists. |
| Sullivan et al [98] | 2023 | USA | Quantitative survey | The objective of this study was to assess the cybersecurity preparedness of United States hospital systems for cyberattack events. Specifically, the researchers surveyed hospital emergency managers to gather data on current preparedness and response procedures related to cybersecurity hazards | The study revealed that American hospitals are currently underprepared for cybersecurity disasters. Specifically: Over half of the surveyed hospitals (52.6%) did not specifically mention cybersecurity in their Emergency Operations Plans (EOPs and Only 24.5% of the surveyed hospital systems had previously activated an emergency response for a cybersecurity incident unrelated to IT failure |
| McEvoy and Kowalski [90] | 2019 | Norway | qualitative, socio-technical risk identification method | The aim is to develop a practical methodology for deriving cybersecurity risks from degraded work practices and human/organizational behavior filling gaps left by traditional technical-focused risk frameworks. The goal is to build “risk narratives” that reveal how behavioral issues contribute to organizational vulnerability. | The method identifies and codes deviations from ideal cybersecurity behaviors across six dimensions: communication, thinking, engagement, investment, planning/processes, and working culture. The method is complementary to technical risk tools and was trailed successfully in defense how poor organizational behavior leads to increased risk. The method is complementary to technical risk tools and was trailed successfully in defense and healthcare sectors, offering a cost-effective and scalable approach for real-world consultancy use. |
| Ewoh and Vartiainen [27] | 2024 | Finland | systematic literature review (SLR) conducted in accordance with PRISMA guidelines | The study aims to explore why healthcare systems are vulnerable to cyberattacks and to propose sociotechnical solutions. It focuses on integrating human, technical, and organizational factors to identify system weaknesses and guide strategic protection measures. | Five core vulnerabilities were identified: human error, lack of investment, legacy systems, digitalization, and complex network-connected devices. It recommends investments in cybersecurity infrastructure, collaboration with educational institutions, adoption of international standards (ISO/IEC 27001), and the development of a sociotechnical development. It recommends investments in cybersecurity infrastructure, collaboration with educational institutions, adoption of international standards (ISO/IEC 27001), and the development of a sociotechnical cybersecurity framework that links technology, people, and processes. |
| Tully et al [41] | 2020 | United States | Qualitative perspective paper | The study aims to highlight the growing threat of cyberattacks in healthcare due to increasing technology integration. It advocates for better quantification tools to assess the impact of these attacks and stresses the need to adapt current U.S. disaster preparedness systems to meet evolving cybersecurity threats. | Healthcare systems are increasingly vulnerable to cyberattacks like WannaCry and targeted ransomware, which cause financial loss, reputational damage, and service disruption. They call for new epidemiologic and scope of cyber threats are outpacing traditional preparedness systems. They call for new epidemiologic research to better understand the clinical and systemic impacts of cyber incidents and to inform the development of targeted mitigation strategies. |
| Arafa et al [42] | 2023 | Japan, Egypt, Saudi Arabia, and the World Health Organization in Switzerland | narrative review that synthesizes peer-reviewed articles and systematic reviews | The review aims to provide a comprehensive overview of emerging digital health technologies like AI, telehealth, blockchain, and IoMT and the cybersecurity risks they introduce. It also outlines best practice frameworks for healthcare institutions to secure digital systems, highlighting education, regulation, and infrastructure readiness. | Emerging technologies such as telemedicine, IoMT, AI, VR/AR, and blockchain enhance healthcare delivery but also introduce significant cybersecurity vulnerabilities, including data breaches, medical device risks, phishing, insider threats, and ransomware. The paper proposes a structured cybersecurity framework that includes access control, encryption, segmentation, staff training, risk assessment, and collaboration across institutions and governments. Ethical, regulatory, and infrastructure challenges remain barriers to secure adoption. |
| He et al [43] | 2021 | United Kingdom and China | scoping review following PRISMA-ScR guidelines | The paper aims to systematically identify the most prominent types of cyberattacks, associated vulnerabilities, and implemented solutions in the healthcare sector during the COVID-19 pandemic. It explores both pandemic-induced threats and inherent cybersecurity weaknesses. | The study review identifies nine major cybersecurity challenges such as poor endpoint management, insecure remote work setups, lack of security awareness, and inadequate incident response and documents 11 categories of solutions, including technical controls, staff training, policy updates, and incident reporting mechanisms. It emphasizes that ransomware, phishing, DDoS, and malware attacks surged during COVID-19, exploiting rapid shifts to remote care and organizational unpreparedness. The authors advocate for long-term strategic planning, improved cyber resilience, root cause analysis of human error, and alignment with frameworks like NIST. |
| Kruse et al [46] | 2017 | United States | systematic review, conducted using PRISMA guidelines | The study aimed to identify cybersecurity trends, including ransomware threats, and examine how these trends impact the healthcare sector. It further sought to evaluate proposed solutions for mitigating these risks by reviewing academic literature. | Healthcare systems lag behind other sectors in cybersecurity preparedness, making them attractive targets for cybercriminals. Two key drivers of vulnerability are rapid technological adoption and underinvestment in security. Medical devices, often integrated into broader networks without robust protection, pose emerging risks. Most breaches stem from human error, with staff training and clearer cybersecurity roles cited as the most effective countermeasures. |
| Pool et al [47] | 2024 | Australia | scoping review and thematic analysis | The paper aims to fill the gap in understanding how and why personal health data breaches occur by contextualizing failures within healthcare organizations. It sets out to identify breach types, causes, and consequences, and to propose an integrated socio-technical model for future research and policy development. | Five primary types of breaches were identified: unauthorized access/disclosure, hacking/IT incidents, improper disposal, loss, and theft. Facilitators include noncompliance, lack of cybersecurity awareness, insecure third-party systems, organizational shortcomings, and weak collective responses. Impacts range from reduced trust and system use to compromised care quality and significant trust repair costs. |
| Calyam et al [49] | 2023 | United States | conceptual framework and technical system design | The goal is to develop an open, scalable, and reusable KGaaS platform to support active cyber defense. The platform aims to help intelligent agents build, query, and maintain cybersecurity-related knowledge across domains like power grids, healthcare, and manufacturing, addressing the limitations of passive, reactive security systems. | The proposed KGaaS enables the construction of domain-specific knowledge graphs for intelligent agents to detect, infer, and respond to cybersecurity threats in real-time. It supports advanced querying, natural language interfaces, integration with LLMs, and threat modelling (e.g., STRIDE) to link cyber threats with operational systems. |
| Messinis et al [50] | 2024 | Greece | systematic review following the PRISMA methodology | The paper aims to bridge a gap in the literature by examining how artificial intelligence (AI) particularly machine learning (ML) and deep learning (DL) can improve the performance and reliability of cybersecurity frameworks for IoMT devices. It reviews key technologies and proposes structured approaches for AI-enhanced cyber defense. | The review identifies how AI technologies contribute to major cybersecurity pillars such as anomaly detection, intrusion detection, blockchain integration, homomorphic encryption, federated learning, and differential privacy. It categorizes common IoMT threats (ransomware, eavesdropping, Sybil attacks, firmware tampering), and showcases how AI enables real-time detection, personalized authentication, and adaptive defense mechanisms. The authors also present multiple AI-enhanced frameworks and highlight future research directions involving lightweight security models and explainable AI. |
| Lopatina et al [51] | 2021 | United Kingdom, Switzerland, and Austria | conceptual, technical analysis paper and a structured risk identification model for IoMT systems | The study aims to identify, classify, and assess the growing cybersecurity risks in cloud-connected Internet of Medical Things (IoMT) systems, especially during and after COVID-19. It proposes a methodology for quantifying these risks and provides guidance on managing data security in distributed sensor networks. | The study identifies extensive vulnerabilities in IoMT systems, such as phishing, ransomware, DDoS, and Sybil attacks. Cloud integration compounds risks like data loss, insider threats, API flaws, and service dependency. A twelve-point risk model is proposed, with high-risk areas including loss of control, provider dependency, and failure to isolate cloud resources. The authors recommend compliance with national cybersecurity standards, continuous IoMT risk reassessment, staff training, pre-emptive budget planning, and cyber insurance. |
| Filipec and Plasilb [52] | 2021 | Czech Republic | Qualitative. applies the Swiss Cheese model | The study aims to extract actionable lessons learned from the cyberattack on a Czech hospital to inform preparedness and crisis response strategies in other healthcare institutions. It explores how organizational, technical, and exposure-level factors interacted to enable the breach and shaped the recovery effort. | The case illustrates how even a moderately secure hospital can be severely impacted by a coordinated ransomware attack. The infection (Emotet → TrickBot → Ryuk) paralyzed hospital operations, forcing a full system rebuild. Despite no ransom being paid and no data loss, the damage exceeded 2 million USD. Key success factors included fast disconnection, strong backup systems, and effective collaboration with Czech cyber authorities (NUKIB). The authors emphasize that updated IT infrastructure, organizational preparedness, segmented networks, and regular training are essential. They also advocate for a proactive “green field” recovery approach and inter-hospital cooperation to share cybersecurity resources and best practices. |
| Wazid et al [53] | 2022 | India and South Korea | comprehensive review article that proposes a generalized security framework for Healthcare 5.0 | The study aims to develop a robust, secure framework for Healthcare 5.0 systems that integrate technologies such as IoT, AI, blockchain, and cloud computing. The objectives include identifying application domains, security threats, comparing existing schemes, and outlining research challenges in deploying secure and scalable smart healthcare systems. | The study presents a layered security model for Healthcare 5.0, addressing threats like DoS, malware, insider attacks, and blockchain-specific vulnerabilities. It identifies the need emphasizes eight, scalable, and interoperable security schemes due to the heterogeneous and resource-constrained nature of smart medical devices. The proposed framework emphasizes authentication, access control, intrusion detection, and blockchain-based data integrity. |
| Ogunniye et al [54] | 2024 | United Kingdom | a review , framework development paper and presents the PETRAS socio-technical framework | The study aims to define and demonstrate a socio-technical research framework that integrates technical innovation with social, ethical, regulatory, and governance requirements in IoT systems. The goal is to support secure, ethical, and publicly accepted deployment of emerging technologies through collaborative, interdisciplinary research. | The PETRAS framework outlines three interacting subsystems technical, social, and external environment that govern IoT cybersecurity development. Key socio-technical requirements include privacy, ethics, trust, reliability, acceptability, and security. Through case studies like P-PITEE, the paper demonstrates participatory methods for involving stakeholders in designing IoT policy and infrastructure. |
| Rehman et al [55] | 2022 | Pakistan, United Arab Emirates, South Korea, Malaysia, Slovakia, Hungary, and Germany | a technical research study and simulation that presents a novel healthcare 5.0 architecture integrating blockchain and federated learning (FL), supported by a RTS-DELM-based Intrusion Detection System (IDS) | The study aims to design a secure, decentralized healthcare monitoring system by combining federated learning, blockchain, and deep extreme learning models. The objectives are to enhance patient data privacy, reduce intrusion risks, and improve disease prediction accuracy in the emerging healthcare 5.0 paradigm. | The proposed system achieved 97% accuracy for Parkinson’s disease prediction and 96.18% accuracy for intrusion detection, outperforming other machine learning models in the literature. It enables real-time monitoring using IoMT sensors, preserves data privacy through federated learning, and ensures system security with blockchain and IDS. The RTS-DELM approach enhances computational efficiency while maintaining accuracy. The study demonstrates that integrating blockchain with FL is a promising direction for scalable, secure, and privacy-preserving smart healthcare systems. |
| Semancik and Wells [56] | 2023 | United States | a technical industry paper, case study and detailed review of cybersecurity vulnerabilities and protection strategies for O-Level (Organizational-Level) military test systems. | The paper aims to identify and mitigate cyber risks in modern automatic test equipment (ATE) systems used at the flight line and depot levels in military settings. It introduces security guidelines, interface-level vulnerabilities, and design-phase recommendations to enhance operational cybersecurity. | Modern O-Level test sets are increasingly vulnerable due to their microprocessor-based designs, operating system dependencies, and use of USB/SD interfaces. Risks include OS-level exploits, physical media misuse, and interface exposure. The paper recommends secure design practices like using a custom OS, implementing STIG guidelines, encrypting sensitive files, and integrating development environments for controlled access. |
| Giansanti [57] | 2021 | Italy | qualitative conceptual overview article. It presents a thematic discussion | The article aims to outline the scope and urgency of cybersecurity in digital health, particularly as it relates to networked medical devices, hospital systems, and data privacy. It emphasizes the need for sector-specific adaptations of cybersecurity strategies and highlights gaps in preparedness, regulation, and training. | The study underscores that cybersecurity in healthcare must encompass technical, operational, and educational domains ranging from data encryption and secure networks to disaster recovery and user training. High-risk sectors include wearable and implantable devices, Picture Archiving and Communication Systems (PACS), and Hospital Information Systems (HIS). Vulnerabilities in these systems can lead to data breaches or patient harm. The author calls for expanded research, regulatory attention, and public awareness, especially regarding citizen-facing mobile apps and digital contact tracing during crises like COVID-19. |
| Lee [58] | 2023 | United States | Mix method a content analytics-based methodology, combining quantitative regression analysis with qualitative thematic analysis | The study aims to systematically identify, classify, and interpret data breach patterns in U.S. healthcare providers using publicly available Health and Human Services (HHS) data. It seeks to understand the relationship between breach types, breach locations, vulnerabilities, and mitigation strategies by mining web-based incident descriptions. | The study finds that critical breach types differ by location: Hacking/IT incidents dominate for email and network servers, Theft is significant for laptops, and Unauthorized Access/Disclosure is prominent in electronic medical records and paper/film. A novel contribution is the integration of quantitative dominance analysis with qualitative theme mapping, offering a practical guide for cybersecurity investment prioritization training, phishing awareness, multi-factor authentication, encryption, and breach notification. A novel contribution is the integration of quantitative dominance analysis with qualitative theme mapping, offering a practical guide for cybersecurity investment prioritization in healthcare. |
| Arora et al [59] | 2014 | United States | a narrative review and policy-focused perspective | The article aims to explore the privacy, security, and confidentiality challenges involved in mobile health (mHealth) research, especially concerning sensitive data like alcohol use. It discusses how mHealth’s potential for real-time intervention and data collection must be balanced against rigorous protections for participants. | The authors emphasize that the promise of mHealth continuous data collection from mobile and wearable devices raises serious risks around data breaches, participant safety, and regulatory compliance. The article outlines key U.S. regulations (HIPAA, Common Rule, 42 CFR Part 2), highlights risks like third-party developer access and low technological literacy, and recommends multi-layered security strategies including encryption, two-factor authentication, remote wiping, and role-based data access. |
| Wang et al [60] | 2015 | China | Mix method a technical security research paper and a novel improved scheme using Chebyshev chaotic maps | The study aims to improve secure authentication for telecare medicine information systems by addressing flaws in an earlier scheme that was vulnerable to user impersonation and lacked anonymity. The goal is to develop a scheme resistant to common attacks while remaining computationally efficient for use in low-power medical devices. | The authors demonstrate that Lin’s scheme fails to protect user anonymity and is vulnerable to impersonation and guessing attacks. Their improved scheme introduces hashed identity masking and random variables, offering mutual authentication, anonymity, and protection against DoS, replay, insider, and man-in-the-middle attacks. While the proposed method adds a slight increase in hash function operations, it maintains comparable performance (98.4 ms total cost) to Lin’s scheme (98.0 ms), making it a secure and practical solution for healthcare systems. |
| Abraham et al [61] | 2019 | United States | a qualitative study using grounded theory with thematic coding | The Study aims to understand why many U.S. healthcare organizations adopt a chaotic or reactive approach to cybersecurity (“muddling through”) and proposes a strategic roadmap for transitioning to a more deliberate, risk-based, and resilient cyber governance model. | The study identifies critical systemic issues: vast attack surfaces due to IoT and mobile devices, fragmented and overlapping security frameworks, ambiguous compliance mandates, immature IT infrastructure, and a lack of executive cyber-risk awareness. It proposes a cybersecurity risk management roadmap focused on risk profile organization-widening, investment valuation, and organizational communication. The authors stress the importance of senior leadership engagement, layered defences, third-party accountability, and cultivating a data-driven cybersecurity culture. The study concludes that cybersecurity must be approached as a strategic, organization-wide priority rather than a purely technical concern. |
| Wasserman and Wasserman [62] | 2022 | United States | a structured scoping review | The study aims to assess whether U.S. hospitals are adequately prepared for cyber threats and to clarify the most critical cybersecurity concerns in clinical settings. The focus is on identifying trends in healthcare cyber risk, clinical technologies used, associated vulnerabilities, and the effectiveness of current protective strategies. | The study review concludes that many U.S. hospitals are severely underprepared for cyberattacks. Key vulnerabilities include outdated operating systems, constant data access reputational, reliance on internet-connected medical devices, weak internal training, and underfunded IT departments. The most frequent threats are phishing, ransomware, DDoS, privilege escalation, and device spoofing. Regulatory frameworks are fragmented, and cyber defenses are often technical but insufficient. A lack of coordination, funding, and cybersecurity literacy among healthcare workers exacerbates the risks. Importantly, the real victims of these attacks are patients, who may suffer reputational, privacy, and even physical harm. |
| Janith et al [63] | 2021 | Sri Lanka | quantitative a technical design and implementation study | The aim is to create a unified, cost-effective cybersecurity system for healthcare institutions that lack dedicated IT security teams. The paper targets vulnerabilities in PHI handling, ransomware defense, automated security hardening, and denial-of-service attacks by developing a scalable, AI-driven security framework tailored for healthcare environments. | SentinelPlus achieved high accuracy across modules: 93.6% in drug/disease classification for email risk scoring, over 97% in ransomware detection, and 99.8% in intrusion detection using random forest classifiers. The system’s automation of STIG-based configuration management through natural language processing significantly reduces labor costs. It proves viable for integration into healthcare IT systems without extensive infrastructure changes, especially benefitting small to medium providers. |
| Dameff et al [64] | 2019 | United States | design and implementation of three high-fidelity clinical simulation scenarios. experimental, simulation-based study | The aim was to develop and test clinical simulation scenarios in which patient harm results from hacked medical devices. The objective was to assess whether emergency physicians could recognize, diagnose, and respond effectively to these novel patient safety threats in real time. | None of the participating physicians identified the cybersecurity compromise as the root cause of patient deterioration in any scenario, despite successfully stabilizing patients clinically. This highlights a critical knowledge gap among clinicians regarding cyber threats in connected medical technologies. The simulations successfully demonstrated that educational interventions using simulation can raise awareness and potentially improve future clinical responses to medical cyber incidents. |
| Feeley et al [65] | 2022 | Ireland | a case study and qualitative practice report | The goal is to describe the clinical, logistical, and operational challenges caused by the ransomware incident and share the adaptive solutions employed to maintain continuity of care in orthopaedic trauma and elective services during the system outage. | The cyberattack crippled access to electronic health records, radiology systems (NIMIS), lab results, and staff communications. In response, hospitals adopted paper-based workflows, secure messaging apps for referrals, hard-copy radiology transport, and deployed portable imaging in outpatient departments. Key lessons include the importance of clinical adaptability, interdepartmental collaboration, and cybersecurity training. |
| Beaman et al [66] | 2021 | Canada and Saudi Arabia | Mix method a comprehensive literature review and experimental research study | The study aims to review state of the art ransomware mitigation techniques, highlight gaps in current solutions, and identify future research directions. It also seeks to validate evasion techniques through empirical testing and propose a taxonomy of ransomware defense strategies. | The study confirms that ransomware threats surged during COVID-19 due to remote work and weaker home network security. The emphasize that ransomware bypassed 8 popular antivirus programs, demonstrating limitations of existing tools. The paper categorizes prevention strategies into access control, key management, data backup, and user awareness; detection methods include file entropy analysis, network traffic inspection, and machine learning. The authors recommend investing in hybrid encryption analysis, fine-grained behavioural modelling, and ransomware honeypots. They emphasize that future research must address advanced ransomware evasion and obfuscation tactics, especially in healthcare and critical infrastructure sectors. |
| Hijji et al [67] | 2021 | Saudi Arabia | a Qualitative Multivocal Literature Review (MLR) that synthesizes findings from both formal peer-reviewed sources and grey literature. | The objective was to identify and categorize social engineering attack types, techniques, platforms, malicious software, and targeted sectors emerging during the COVID-19 pandemic. The authors aimed to expose key cybersecurity challenges, economic impacts, and propose future solutions using emerging technologies like AI, blockchain, and big data analytics. | Phishing (35%) was the most used technique, followed by spam, scams, smashing, and vishing. Socio-technical attacks dominated (44%), exploiting platforms like fake emails, websites, and mobile apps. Hospitals and healthcare systems were the most targeted sectors. The most common malware included ransomware ( Maze, Netwalker), trojans (Emotet), and bots (Loki-Bots). Economic losses were massive, with institutions like UCSF paying over $1 million in ransoms. The paper highlights challenges such as remote work vulnerabilities, poor privacy governance, and weak security training. It proposes AI, blockchain, training, and big data analytics as pathways to resilience. |
| Fernando et al [68] | 2014 | Australia | a qualitative, empirical case study integrated with a thematic literature review. Socio-Technical-Material (STM) framework | The study aims to explore and define a Natural Hospital Environment (NHE) through a Socio-Technical-Material (STM) framework, assessing how systemic, regulatory, architectural, and technological challenges impact clinicians’ ability to maintain privacy and security (P&S) in e-health practices. The objective is to inform more realistic and sustainable design and policy decisions for hospital-based digital health systems. | The study finds that clinicians face persistent STM challenges including outdated infrastructure, shared workspaces, inconsistent training, slow systems, and cumbersome authentication. These barriers often force staff to circumvent formal P&S protocols (e.g., password sharing, printing handover sheets). Privacy risks such as aural leaks and screen exposure are frequent due to spatial design. The authors argue that STM-informed frameworks better capture the real limits clinicians face in securely using e-health systems and recommend redesigning hospital environments and policies to reflect these constraints for meaningful cybersecurity improvement. |
| Pranggono and Arabo [69] | 2021 | United Kingdom | a thematic analysis and review studies | The study aims to highlight and analyze how the COVID-19 pandemic increased the risk and frequency of cyber-attacks, particularly in the healthcare sector and in work-from-home settings. It also outlines practical mitigations and policy implications to better protect data, systems, and people. | The paper categorizes pandemic-era cyberattacks into phishing, malware, and DDoS, with healthcare organizations being prime targets. Cybersecurity must be framed not only as a technological issue but as a holistic organizational key vulnerability include outdated systems, insecure home networks, and lack of training. Mitigation strategies include VPN use, multi-factor authentication, device patching, anti-malware, network segmentation, and physical security. It strongly advocates for AI-powered intrusion detection systems, and for healthcare to adopt comprehensive cybersecurity governance models like CERT-RMM. Cybersecurity must be framed not only as a technological issue but as a holistic organizational and process-based challenge. |
| Wilner et al [70] | 2022 | Canada | a scholarly policy analysis and case study review | The study aims to elevate the discourse on cybersecurity in Canadian healthcare by framing it as a national security issue. It investigates the reasons why the healthcare sector is a high-risk target, outlines common attack types and malicious actors, and draws lessons from global case studies to propose better strategies for cyber resilience in Canada. | The authors find that Canada’s healthcare cybersecurity landscape suffers from chronic underfunding, uneven regional capabilities, lack of coordinated national policy, and an institutional culture that under-prioritizes cybersecurity. The study identifies healthcare-specific threats such as ransomware, medjacking, DDoS, and data manipulation, and analyzes attack case studies from Boston Children’s Hospital (DDoS), Anthem (data breach), and the NHS (WannaCry ransomware). It concludes by recommending Canada adopt sector-specific cybersecurity bodies and integrated national frameworks, as seen in Australia’s Digital Health Cybersecurity Centre, New Zealand’s cyber response plan, and specialized CERTs in Norway and the Netherlands. |
| DeFord [71] | 2022 | United States | a policy and opinion article with strategic guidance | The article aims to convince healthcare leaders that digital transformation is inseparable from cybersecurity transformation. It advocates for recognizing cyber threats as adversarial and sophisticated, not merely technical disruptions, and promotes proactive strategy grounded in threat intelligence. | Cybersecurity must evolve alongside healthcare’s digital infrastructure to ensure safe, sustainable care delivery. The author argues that viewing cybersecurity merely as protection analyzing malware or ransomware is insufficient. Instead, health systems must adopt a threat-informed approach, analyzing the motivations, tools, and structures of adversaries. |
| Gordon et al [73] | 2019 | United States | a quantitative retrospective observational study | The study aimed to determine whether a targeted, mandatory online phishing awareness program could reduce click rates among “high-risk” employees defined as those who clicked on at least five phishing simulations compared to those at lower risk. | The study found that while phishing clicks rates decreased overall across repeated campaigns, the mandatory training had little impact on reducing phishing susceptibility among high-risk employees. Those who underwent the training remained significantly more likely to click on phishing emails, with click rates ranging from 10% to 25% even post-intervention. The findings suggest that current online training may be insufficient and that more effective or tailored interventions (in-person or real-time training) are needed to protect healthcare institutions from phishing-related threats. |
| Abbou et al [82] | 2024 | Israel | a Qualitative retrospective observational case study | The study aims to document the clinical impact of a full-scale hospital cyberattack and to identify which digital system recoveries (EMR, imaging) are most critical to restoring hospital functions. | Despite the cyberattack, procedures like births and catheterizations continued; however, there was a sharp decline in surgeries, ER visits, and hospital occupancy, particularly during the first week post-attack. Clinical activity increased steadily as systems were restored, with a 30% increase following EMR and lab recovery, and a 50% surge after imaging systems came back online. The study concludes that restoring digital infrastructure especially EMRs and diagnostics is essential to operational recovery, and recommends all hospitals develop clear cyber crisis protocols to ensure continuity of care under similar disruptions. |
| Harrison et al [83] | 2022 | United States | a qualitative retrospective incident report and case study | The objective of the study was to detail the emergency response, recovery process, and lessons learned following a cybersecurity incident that compromised a radiation therapy system critical to patient care. | The RVS shutdown led to a complete halt in radiation therapy for the first 24 hours. Through emphasizes hospital EMRs, direct DICOM transfers, and paper charting, the department resumed 50% of treatments within 48 hours and 95% within a week. Full RVS functionality was restored in 4.5 weeks. Key lessons included the importance of system backups, dry-run training, consistent vendor standards, and a structured recovery plan. The case emphasizes that resilience in radiation oncology requires technical, procedural, and human capital readiness to maintain safe care delivery during prolonged IT outages. |
| Mohammed [84] | 2022 | Trinidad and Tobago | a conceptual and case-based study, grounded in stakeholder theory, using qualitative case analysis of three high-profile data breaches (Anthem, Equifax, and Citrix) to propose a framework of four key recovery areas | The objective is to provide a structured theoretical model for post-data breach recovery, identifying stakeholder-aligned strategies across various dimensions to guide both organizational response and future academic research in cybersecurity recovery. | The study identifies four core areas critical to effective recovery from data breaches: customer recovery, employee recovery, process recovery, and regulatory recovery. The author emphasizes that focusing solely on technical fixes overlooks broader impacts on internal and external stakeholders. The Data Recovery Areas Model offers organizations a roadmap for restoring trust, maintaining compliance, and regaining operational integrity post-incident. It also serves as a foundation for future empirical studies into breach recovery best practices. |
| Keogh et al [85] | 2024 | Ireland | Mix-method. a retrospective case study supported by quantitative data, qualitative stakeholder interviews, and reflective essays | The study aims to analyze the full-scale operational impact of a ransomware cyberattack on cancer services in a national hospital, and to derive recommendations for improving cyber-preparedness and patient safety in future healthcare incidents. | The cyberattack caused the complete shutdown of IT systems, halting cancer surgeries, radiotherapy, outpatient clinics, and diagnostic services. Radiology, lab, and pathology suffered dramatic drops in capacity. Patient care emphasizing emergency protocols like manual data handling, paper charting, and outsourcing to private hospitals. Full system restoration took months, with data backlogs, communication outages, and treatment delays, especially in radiation oncology. The psychological toll on patients and staff was significant. The study presents a ten-step institutional guide for handling future cyberattacks, emphasizing the need for resilient systems, cross-site backups, and staff training to ensure continuity of care. |
| Bhuyan et al [86] | 2020 | United States | a comprehensive narrative review and policy recommendation article, synthesizing current literature and public health cybersecurity incidents. | The study aims to transition healthcare cybersecurity from a reactive, fragmented posture to a proactive, strategic model. It does this by identifying the types of cyberattacks, the key players involved (attackers, defenders, developers, and end users), and formulating policy-level and organizational recommendations to improve systemic security resilience. | Cybersecurity threats in healthcare ranging from ransomware and DoS to SQL injection and phishing are rising and multifaceted. Attackers include cybercriminals, hacktivists, and insiders, while vulnerabilities span technical and human dimensions. The paper advocates for a risk-based, layered approach to defense, including resilient governance structures, cyber insurance, standardized development protocols, and integration of IT strategy with enterprise risk management. |
| He et al [87] | 2022 | United Kingdom and China | a methodological and case study-based paper that proposes a proactive cybersecurity incident response (IR) framework | The objective is to transform traditional reactive IR strategies into proactive systems by embedding CTI at every stage of the IR lifecycle. The authors aim to fill a significant gap in healthcare cybersecurity where CTI is underutilized, and they seek to offer a comprehensive, implementable model for real-world healthcare providers. | The study identifies that traditional IR processes in healthcare are largely reactive and fragmented, limiting their capacity to prevent or mitigate threats like ransomware. The proposed CTI-informed IR model enhances preparedness, detection, analysis, containment, recovery, and post-incident activities. Applied to the NHS WannaCry case, the model exposes several missed opportunities, such as lack of early patching (MS17-010), unclear IR plans, and underutilized threat advisories. The study demonstrates that integrating CTI from platforms like US/UK CERT, MITRE, and MISP significantly improves detection accuracy, containment timing, and recovery outcomes. It recommends embedding these practices into national healthcare cybersecurity strategies and IR tools like SIEM and SOAR systems. |
| Jalali et al [88] | 2019 | United States | a systematic literature review using EARS framework | The objective was to bridge the knowledge gap in healthcare cybersecurity by providing evidence based, actionable strategies for incident response. The authors aim to help healthcare leaders and IT professionals design robust response protocols to handle cyberattacks efficiently and ethically. | The authors develop the EARS framework (Eight Aggregated Response Strategies), grouped into pre- and post-incident phases. Pre-incident strategies include building an incident response plan, HITRUST), but emphasizes security policies, involving key personnel, and conducting regular mock recovery tests. Post-incident actions involve technical containment, investigation and documentation, embedding ethics and external stakeholder communication, and implementing a damage assessment and recovery algorithm. The study concludes that healthcare organizations are significantly underprepared for cyberattacks and must adopt proactive, structured, and ethical response models. The framework aligns partially with industry standards (NIST, ISO, HITRUST), but emphasizes healthcare-specific challenges. |
| Tin et al [91] | 2023 | United States | a Mex-method descriptive quantitative and qualitative analysis | The objective is to provide healthcare professionals especially in emergency medicine with a foundational understanding of cyberthreats, including the scale, trends, and vulnerabilities within the healthcare sector. The goal is to increase readiness and resilience through data-driven insight. | From 2011 to 2021, there were 4026 breaches affecting over 303 million individuals in the U.S. Healthcare providers accounted for 74% of breaches. The top threats were hacking/IT incidents (43%), unauthorized access (27%), and theft (21%). Network servers and email were the most common breach entry points. Emergency departments, with their critical role in hospital operations, were highlighted as especially vulnerable and in need of proactive cybersecurity protocols. The paper emphasizes the urgent need for healthcare-specific cybersecurity training, incident response plans, and better infrastructure management, especially for legacy systems and IoT medical devices. It advocates for a proactive, system-wide cybersecurity culture supported by government regulation and continuous clinician education. |
| Offner et al [11] | 2020 | Australia | a systematic review following PRISMA guidelines | The primary goal is to assess cybersecurity capability in Australian healthcare organisations, evaluate trends and threats associated with electronic health record (EHR) systems, and offer strategies to mature cybersecurity infrastructure and culture. It specifically examines how Australia’s My Health Record implementation compares to global developments. | The review finds that Australian healthcare organisations lag behind other sectors in cybersecurity preparedness, with a lack of skilled professionals, poor cyber-hygiene, and outdated IT infrastructure. Cybersecurity risks are intensified by the integration of legacy systems with new digital tools, increased reliance on IoT devices, and growing use of health apps. Major threat categories include malware, ransomware, phishing, DDoS attacks, and insider threats. The authors highlight that current mitigation efforts are largely reactive. They advocate for holistic strategies involving proactive culture change, updated regulation, better education and training, and adoption of international standards like NIST and ISO. A key contribution is the mapping of themes including emerging threats, mitigation measures, and Australia-specific challenges. |
| Patel and Makaryus [93] | 2024 | United States | a narrative review and expert commentary | The study aims to assess the cybersecurity risks associated with cardiac digital endpoints such as implants, wearable devices, and AI-based systems and outlines the roles and responsibilities of policymakers, device manufacturers, and patients in ensuring data safety and integrity in the growing field of digital cardiology. | As reliance on remote monitoring grows, so does the risk of cybersecurity breaches affect cardiac devices. The authors stress that patient safety depends on secure data transmission and access control. Key recommendations include: the development of prioritize security policies, secure communication protocols, and greater investment in digital literacy for patients. Policymakers must implement clear guidelines to govern access and ensure device-level encryption and network security, while manufacturers must prioritize security at the design stage. |
| Coventry et al [80] | 2020 | Ireland, Italy, and Greece | a qualitative, multi-site, thematic analysis design | The aim is to identify the behavioural drivers and systemic barriers contributing to insecure cybersecurity practices in healthcare. The study explores the human and environmental factors influencing these behaviours to inform practical and policy-level interventions. | The study uncovers widespread insecure behaviours, including weak password practices, shared login credentials, unsafe email and USB use, lack of encryption, and insecure remote access. These are driven by four main facilitator standardized password policies, better HCI design, targeted training, secure mobile platforms, and policy enforcement emphasizing is often seen as a barrier to patient care, and many behaviours are rationalized to improve workflow efficiency. Staff feel undertrained, unsupported, and excluded from policy processes. The authors recommend a suite of reforms standardized password policies, better HCI design, targeted training, secure mobile platforms, and policy enforcement emphasizing that behavioural and cultural change is as vital as technical solutions. |
| Grande et al [95] | 2020 | United States | a qualitative study using in-depth, semi structured interviews | The goal is to explore and describe privacy challenges emerging from digital health data footprints, focusing on how non-traditional digital interactions (like social media use or GPS data) contribute to health-related inferences, often without consumer awareness or adequate regulation. | the study reveals that all digital data regardless of source can become health data when aggregated, posing serious privacy and policy concerns. Experts emphasized five core risks: invisibility (users are unaware data are collected), inaccuracy (digital data can misrepresent health status), immortality (data persist indefinitely and are often resold), marketability (health-related digital data hold high commercial value), and identifiability (individuals can be reidentified through data linkages). |
| Parmeggiani et al [94] | 2024 | Italy | Review | To determine the best security standards to use and compare them to define a correct cybersecurity framework | The ISO 27799 family’s standard provides right additional directives that are covered in part from GDPR and this combination is the best |
| Alhassani et al [97] | 2024 | United Kingdom | Scoping review | To identify what is already about the barriers and drivers that influence HCP’s compliance or intention to comply with their local data privacy policies, as these relate to the EHR. To identify the theoretical frameworks that have been applied to this area of study and how these relate to different field of study. To synthesize the information gained regarding barriers and drivers that influence the HCP’s compliance or intention to comply with their local data privacy policies into one holistic framework. | For an organization to develop approaches that improve compliance, they must take a multifaceted approach working to improve training, capability, and training of their workforce and also ensuring that organizational culture is one that is mature in its management of change, has sufficient structural resources and clearly values individuals for their work in this area. |
| Sekandi et al [75] | 2022 | Uganda | Scoping review | To explore ethical, legal, sociocultural, and regulatory concerns from the perspective of key holders in the public and private sectors in Uganda. | Sociocultural issues seem to influence the different levels of positive and negative perceptions around data privacy and confidentiality concerns by gender and user group across the East African region. mHealth data have the potential to enhance or jeopardize privacy. The lack of  Comprehensive ethical framework to guide the use of mHealth in public health research and practice remains a challenge across countries in the East African Community (EAC) region. Legal regulation of personal health information with EAC is not uniformly developed. |
| Sari et al [79] | 2022 | Indonesia | Systematic literature review | To investigate the research trend and antecedent factors of information security behavior in the health care context involving various types of HIS users in health care organizations, including clinical staff, non-clinical staff, and patients | Health care provider other than hospital are understudied in the context of information security behavior. Information security risks come not only from internal users at the health care provider but also from external users who have access rights to the system. Most studies indicated that organizational factors significantly impact security behavior, mediated by individual factors. |
| Zhan et al [9] | 2024 | Pakistan | Quantitative - Purposive sampling technique | To examine the impact of the factors related to cybersecurity influencing the adoption of the health information system | The study found that external attacks and technological factors have a significant impact on acting as a barrier to the adoption of information system in the health care industry, while employee or personal factors, such as misuse or a lack of skills, have no significant impact on acting as a barrier to the adoption of the information system in health care. |
| Lohrke and Frownfelter-Lohrke [89] | 2023 | United States | Review | To chart a path forward for management scholars to study cybersecurity issues, including how incidents affect companies from the front line to executive suite | The review show that cybersecurity event research fertile ground for additional theory development and texting in important management research area ranging from employee training to cross management. Also, scholars have examined cybersecurity events from four major perspectives-antecedents of, reactions to, and outcome from cybersecurity threats, as well as potential moderators of this process. |
| Alfawzan [77] | 2022 | Switzerland | Scoping review and content analysis | To assess the privacy policies, data sharing, and security policies of women’s mHealth apps on the current international market (the APP store on the Apple operating system [iOS] and Google Play on the Android system) | The study found that the most popular women’s mHealth apps on the market have poor data privacy, sharing, and security practices. Also, most apps provided their privacy policies only in English. |
| Ireland et al [74] | 2019 | United Kingdom | Mixed methods -Systematic literature review and principal component analysis | To explore physical and environmental factors and their role in predicting security incidents in high secure psychiatric care | Findings revealed that staff failure to value patients and show respect was associated with incidents supports the theory that procedural justice and legitimacy of authority can influence behavior. The fairness patients perceived in relation to staff behavior was also related to incidents, and mediated the relationship between interpersonal style and  aggressive incidents. |
| Monteith et al [78] | 2021 | United States | Narrative review | To examine the changing use of technology, societal impacts of the pandemic, how cybercrime is evolving, individual vulnerabilities to cybercrime, and special concerns for those with mental illness | Human factors are a central component of cybersecurity as individual behaviour, personality traits, online activities, and attitudes to technology impact vulnerability |
| Cartwright [48] | 2023 | United Arab Emirates | Review | To examine and highlight the cybersecurity challenges and vulnerabilities in health care sector. | The study found that the health sector is underfunding cybersecurity, leading to outdated equipment, software, and reduced IT staff. |
| Hines et al [72] | 2023 | United States | Mix method a national survey of plastic surgeons with a scoping literature review | The goal was to assess cybersecurity awareness, practices, and barriers in plastic surgery, a field uniquely vulnerable due to sensitive patient imaging and data. It also aimed to identify gaps in existing guidelines and offer recommendations to mitigate cyber risks. | Among 69 plastic surgeons who completed the survey, over 85% acknowledged cybersecurity as a critical concern, yet reported barriers such as lack of expertise (41.7%), funding, and time. Older surgeons (55+) were significantly more likely to have preventive technology and formal policies in place. The literature review found no articles directly addressing cybersecurity in plastic surgery, though 12 healthcare-related cybersecurity articles were reviewed. Key vulnerabilities identified include outdated software, weak staff cyber hygiene, and lack of security awareness training. |
|  |  |  |  |  |  |
| Kaberuka and Johnson [7] | 2020 | Rwanda | System-Theoretic Accident Modeling Process (STAMP). with NIST control taxonomy and ethnographic techniques, interviews, and document analysis | The study aims to address socio-technical cyber security challenges in healthcare systems of emerging nations, with a particular focus on improving cyber-security in a Rwandan hospital's Picture Archiving and Communication System (PACS). | The study reveals that open-ended analytical technique requires additional methodological structure in countries where there are significant shortages of trained analysts; to guide the application of STPA-sec and also to provide common reference |
| Yeng et al [76] | 2021 |  | Literature review and Healthcare Security Practice Analysis, Modelling, and Incentivization (HSPAMI) project | The aims of this study were to develop a comprehensive framework for modeling and analyzing health care professionals’ information security practices related to their individual characteristics, such as their psychological, social, and cultural traits | The key finding was the development of a comprehensive framework called the psychosociocultural (PSC) framework for modelling and analyzing healthcare professionals' information security practices. This framework incorporates psychological, social, cultural, and demographic variables to provide a holistic approach to understanding and addressing security issues in healthcare |
| Branley-Bell et al [81] | 2020 | Ireland, Italy, and Greece | Qualitative Study | The study introduces and apply the AIDE approach (Assess, Identify, Develop, Evaluate) for eliciting positive cybersecurity behaviours among healthcare staff. | The study finds that AIDE approach can effectively identifies and addresses behavioural vulnerabilities in healthcare cybersecurity.  Furthermore, they authors noted that Behaviour change interventions, when systematically applied, can lead to improved cybersecurity practices among healthcare staff. |
| Al-Qarni [92] | 2023 | Bisha-Saudi Arabia | Qualitative Review Literature | The study reviewed and analysed recent cyberattacks of healthcare systems and proposed mitigation strategies | Lack of preparedness: Many healthcare organizations lack comprehensive security policies, leaving them vulnerable to attacks |
|  |  |  |  |  |  |
