# Supplementary material for: Sociotechnical Cybersecurity Framework for Securing Health Care From Vulnerabilities and Cyberattacks: Scoping Review
Source: J Med Internet Res. 2025 Oct 15;27:e75584. doi: 10.2196/75584 (PMC12572753; doi:10.2196/75584)
Supplement: Multimedia Appendix 4 [file jmir_v27i1e75584_app4.docx]

**Table S1.** Critical appraisal of qualitative studies.

| Group 1: Study design and methodology | | | | | | Group 2: Researcher influence | | Group 3: Participants and their voices | Group 4: Ethical approval | Group 5: Interpretation of the article results |  |
| --- | --- | --- | --- | --- | --- | --- | --- | --- | --- | --- | --- |
| Study (Author, Year) | Q1: Congruity between philosophy & methodology | Q2: Congruity between methodology & research question | Q3: Congruity between methodology & data collection | Q4: Congruity between methodology & data analysis | Q5: Congruity between methodology & interpretation | Q6: Researcher position considered culturally or theoretically? | Q7: Is the influence of the researcher on the research) | Q8: Representation of participants and their voices | Q9: Ethical approval reported | Q10: Conclusions flow from analysis/data | Overall Quality |
| Kaberuka and Johnson [6] 2023 | Yes | Yes | Yes | Yes | Yes | Yes | Yes | No | No | Yes | High |
| McEvoy and Kowalski [90] 2019 | Yes | Yes | Yes | Yes | Yes | No | Yes | Yes | No | Yes | High |
| Abraham et al. [61] 2019 | Yes | Yes | Yes | Yes | Yes | Yes | Yes | Yes | No | Yes | High |
| Dameff et al. [64] 2019 | Yes | Yes | Yes | Yes | Yes | No | Yes | Yes | No | Yes | High |
| Fernando et al. [68] 2014 | Yes | Yes | Yes | Yes | Yes | No | Yes | Yes | No | Yes | High |
| Wilner et al [70] 2022 | Yes | Yes | Yes | Yes | Yes | No | N/A | No | No | Yes | High |
| Harrison et al [83] 2022 | Yes | Yes | Yes | Yes | Yes | No | Yes | Yes | No | Yes | High |
| Mohammed [84] 2022 | Yes | Yes | Yes | Yes | Yes | No | Yes | Yes | No | Yes | High |
| He et al [87] 2022 | Yes | Yes | Yes | Yes | Yes | No | No | No | No | Yes | Medium |
| Jalali et al [88] 2019 | Yes | Yes | Yes | Yes | Yes | No | No | No | No | Yes | Medium |
| Coventry et al [80] 2020 | Yes | Yes | Yes | Yes | Yes | No | Yes | Yes | Yes | Yes | High |
| Grande et al [95] 2020 | Yes | Yes | Yes | Yes | Yes | No | Yes | Yes | Yes | Yes | High |
| Parmeggiani et al [94] 2024 | Yes | Yes | Yes | Yes | Yes | No | No | No | No | Yes | Medium |
| Alhassani et al [97] 2024 | Yes | Yes | Yes | Yes | Yes | yes | yes | yes | yes | Yes | High |
| Sekandi et al [75] 2022 | Yes | Yes | Yes | Yes | Yes | yes | yes | yes | yes | Yes | High |
| Alfawzan [77] 2022 | Yes | Yes | Yes | Yes | Yes | yes | yes | yes | No | Yes | High |
| Yeng et al [76] 2021 | Yes | Yes | Yes | Yes | Yes | yes | yes | yes | yes | Yes | High |
| Branley-Bell et al [81] 2020 | Yes | Yes | Yes | Yes | Yes | Yes | Yes | Yes | Yes | Yes | High |
| Kaberuka and Johnson [7] 2020 | Yes | Yes | Yes | Yes | Yes | Yes | Yes | No | Yes | Yes | High |

Rating Guide: 8 -10 Yeses = High Quality; 5-7 Yeses = Medium; 0 – 4 Yes = Low

**Table S2.** JBL systematic and scoping reviews appraisal tool.

| Systematic reviews | Q1: Is the review question clearly and explicitly stated? | Q2: Were inclusion criteria appropriate for the review questions? | Q3: Was the search strategy appropriate? | Q4: Were sources and resources used to search for studies adequate? | Q5: Were the criteria for appraising studies appropriate? | Q6: Was critical appraisal conducted by two or more reviewers? | Q7: Were methods to minimize errors in data extraction used? | Q8: Were the methods used to combine studies appropriate? | Question 9: Was the likelihood of publication bias assessed? | Q10: Were recommendations for policy and/ or practice supported by the reported data? | Q11: Were the specific directives for new research appropriate? | Final Grading |
| --- | --- | --- | --- | --- | --- | --- | --- | --- | --- | --- | --- | --- |
| Khando [96] 2021 | Yes | Yes | Yes | Yes | Yes | Yes | Yes | Yes | Yes | Yes | Yes | High |
| Lohrke and Frownfelter-Lohrke [89] 2023 | Yes | Yes | Yes | Yes | No | Yes | Yes | Yes | No | Yes | Yes | High |
| Kruse et al [46] 2017 | Yes | Yes | Yes | Yes | Yes | Yes | Yes | Yes | Yes | Yes | Yes | High |
| Hijji et al [67] 2021 | Yes | Yes | Yes | Yes | Yes | Yes | Yes | Yes | No | Yes | Yes | High |
| Ewoh and Vartiainen [27] 2024 | Yes | Yes | Yes | Yes | Yes | Yes | Yes | Yes | Yes | Yes | Yes | High |
| Offner et al [11] 2020 | Yes | Yes | Yes | Yes | Yes | Yes | Yes | Yes | Yes | Yes | Yes | High |
| Dias et al [4] 2021 | Yes | Yes | Yes | Yes | Yes | No | Yes | Yes | No | Yes | Yes | High |
| Jalali et al [104] 2018 | Yes | Yes | Yes | Yes | Yes | Yes | Yes | Yes | Yes | Yes | Yes | High |
| Sari et al [79] 2022 | Yes | Yes | Yes | Yes | Yes | Yes | Yes | Yes | No | Yes | Yes | High |
| Argaw et al [18] 2019 | Yes | Yes | Yes | Yes | N/A | N/A | Yes | N/A | N/A | Yes | Yes | Medium |
| He et al [43] 2021 | Yes | Yes | Yes | Yes | N/A | Yes | Yes | N/A | N/A | Yes | Yes | High |
| Svandova and Smutny [26] 2024 | Yes | Yes | Yes | Yes | N/A | Yes | Yes | N/A | N/A | Yes | Yes | High |
| Pool et al [47] 2024 | Yes | Yes | Yes | Yes | N/A | N/A | Yes | Yes | N/A | Yes | Yes | High |
| Wasserman and Wasserman [62] 2022 | Yes | Yes | Yes | Yes | N/A | Yes | Yes | Yes | N/ A | Yes | Yes | High |
|  |  |  |  |  |  |  |  |  |  |  |  |  |

Rating Guide: 8-10 Yeses = High Quality; 5-7 Yeses = Medium; 0 – 4 Yeses = Low

Not Applicable N/ A = meaning not applicable are scoping review articles regarding bias, and appraisal part aspect of the study.

**Table S3.** MMAT critical appraisal tool for quantitative, mix method and survey.

| Mix Method Studies | Q1 | Q2 | Q3 | Q4 | Q5 | Quality |
| --- | --- | --- | --- | --- | --- | --- |
| Malatji et al [25] 2020 | Yes | Yes | Yes | Yes | Yes | High |
| Szczepaniuk and Szczepaniuk [21] 2023 | Yes | Yes | Yes | Yes | Yes | High |
| Lee [58] 2023 | Yes | Yes | Yes | Yes | Yes | High |
| Wang et al [60] 2015 | Yes | Yes | Yes | Yes | Yes | High |
| Keogh et al [85] 2024 | Yes | Yes | Yes | Yes | Yes | High |
| Tin et al [91] 2023 | Yes | Yes | Yes | Yes | Yes | High |
| Ireland et al [74] 2019 | Yes | Yes | No | No | Yes | Medium |
| Hines et al [72] 2023 | Yes | Yes | Yes | Yes | Yes | High |
| Beaman et al [66] 2021 | No | Yes | Yes | Yes | Yes | Medium |
| Giansanti and Monoscalco [99] 2021 | Yes | Yes | Yes | Yes | Yes | High |
|  |  |  |  |  |  |  |

Rating Guide: High quality = 5 yeses; Medium quality = 3- 4 yeses, Low quality = 0-2 yeses

**Table S4.** Critical Appraisal of Survey and Quantitative (CEBM).

| Survey and quantitative | Q1 | Q2 | Q3 | Q4 | Q5 | Q6 | Q7 | Q8 | Q9 | Q10 | Q11 | Quality |
| --- | --- | --- | --- | --- | --- | --- | --- | --- | --- | --- | --- | --- |
| Garcia-Perez et al [20] 2023 | Yes | yes | yes | Yes | No | Yes | Yes | Yes | Yes | Yes | Yes | High |
| Gordon et al [73] 2019 | Yes | No | Yes | Yes | Yes | Yes | No | Yes | Yes | Yes | Yes | High |
| Zhan et al [9] 2024 | Yes | No | No | Yes | Yes | Yes | Yes | Yes | Yes | Yes | No | Medium |
| Sullivan et al [98] 2023 | Yes | No | No | Yes | Yes | Yes | Yes | Yes | Yes | Yes | No | Medium |
| Rehman et al [55] 2022 | Yes | Yes | Yes | Yes | No | Yes | Yes | Yes | Yes | No | Yes | High |
| Janith et al [63] 2021 | Yes | Yes | Yes | Yes | No | No | Yes | Yes | Yes | No | Yes | Medium |
| Feeley et al [65] 2022 | Yes | Yes | Yes | Yes | Yes | No | Yes | Yes | Yes | No | Yes | High |
| Abbou et al [82] 2024 | Yes | Yes | Yes | Yes | Yes | Yes | Yes | Yes | Yes | No | Yes | High |

Rating Guide: High quality = 10 -11Yeses; Medium quality = 7- 9 Yeses, Low quality = 0-4 Yeses

Scale for the assessment of narrative review articles (SANRA)

**Table S5.** Appraisal for Narrative Review Studies (SANRA).

| Narrative review | Q1: Justification of the article’s importance | Q2: Clear formulation of objectives/ questions | Q3: Comprehensive description of literature search | Q4: Referencing Structured Presentation | Q5: Scientific reasoning | Q6: Appropriate presentation of data | Quality |
| --- | --- | --- | --- | --- | --- | --- | --- |
| Arafa et al [42] 2023 | 2 | 2 | 1 | 2 | 2 | 2 | 11/12 High |
| Messinis et al [50] 2024 | 2 | 2 | 1 | 2 | 2 | 2 | 11/2 High |
| Arora et al [59] 2014 | 2 | 1 | 1 | 2 | 2 | 1 | 9/12 Medium |
| DeFord [71] 2022 | 2 | 1 | 1 | 2 | 2 | 1 | 9/12 Medium |
| Monteith et al [78] 2021 | 2 | 2 | 1 | 2 | 2 | 1 | 10/12 High |
| Cartwright [48] 2023 | 2 | 1 | 1 | 2 | 2 | 1 | 9/12 Medium |
| Bhuyan et al [86] 2021 | 2 | 1 | 1 | 2 | 2 | 1 | 9/12 Medium |
| Coventry and Branley [16] 2018 | 2 | 2 | 2 | 2 | 2 | 1 | 11/12 High |
| Kioskli et al [1] 2021 | 2 | 1 | 1 | 2 | 2 | 1 | 9/12 Medium |
| Alhammad et al [44] 2022 | 2 | 1 | 1 | 2 | 2 | 1 | 9/12 Medium |
| Vukotich, G. [22] 2023 | 2 | 1 | 1 | 2 | 2 | 1 | 9/12 Medium |
| Tully et al [41] 2020 | 2 | 1 | 1 | 2 | 2 | 1 | 9/12 Medium |
| Giansanti [57] 2021 | 2 | 1 | 1 | 2 | 2 | 1 | 9/12 Medium |
| Kandasamy et al [45] 2022 | 2 | 2 | 1 | 2 | 2 | 2 | 11/12 High |
| Filipec and Plasilb [52] 2021 | 2 | 2 | 1 | 2 | 2 | 1 | 10/12 High |
| Al-Qarni [92] 2023 | 2 | 2 | 1 | 2 | 2 | 1 | 10/12 High |
| Semancik and Wells [56] 2023 | 2 | 1 | 0 | 0 | 0 | 1 | 4//12 Low |
| Pranggono and Arabo [69] 2021 | 2 | 1 | 1 | 2 | 2 | 2 | 10/12 High |
| Lopatina et al [51] 2021 | 2 | 1 | 1 | 2 | 2 | 1 | 9/12 Medium |
| Calyam et al [49] 2023 | 2 | 2 | 2 | 1 | 1 | 1 | 9/12 Medium |
| Wazid et al [53] 2022 | 2 | 2 | 1 | 2 | 2 | 1 | 10/12 High |
| Ogunniye et al [54] 2024 | 2 | 2 | 1 | 2 | 2 | 1 | 10/12 High |
| Patel and Makaryus [93] 2024 | 2 | 1 | 1 | 2 | 2 | 1 | 9/12 Medium |
| Zimmermann and Renaud [23] 2019 | 2 | 2 | 1 | 2 | 2 | 2 | 11/12 High |
| Malatji et al [17] 2019 | 2 | 2 | 1 | 2 | 2 | 1 | 10/12 High |
| Sittig and Singh [12] 2016 | 2 | 2 | 1 | 2 | 2 | 1 | 10/12 High |
|  |  |  |  |  |  |  |  |

Rating Guide: High quality = 10- 12 points; Medium quality = 5- 9 points, Low quality = 0-4 0 = Not at all
