# Supplementary material for: Sociotechnical Cybersecurity Framework for Securing Health Care From Vulnerabilities and Cyberattacks: Scoping Review
Source: J Med Internet Res. 2025 Oct 15;27:e75584. doi: 10.2196/75584 (PMC12572753; doi:10.2196/75584)
Supplement: Multimedia Appendix 5 [file jmir_v27i1e75584_app5.docx]

**Quality Appraisal Groups by Study Methodology**

| **Qualitative**-**Joanna and Briggs Tool**    **Group 1: Study design and methodology**  Q1. Is there congruity between the stated philosophical perspective and the research methodology?  Q2. Is there congruity between the research methodology and the research question or objectives?  Q3. Is congruity between the research methodology and the methods used to collect data?  Q4. Is there congruity between the research methodology and the representation and analysis of data?  Q5. Is there congruity between the research methodology and the interpretation of results?  **Group 2: Researcher influence**  Q6. Is there a statement locating the researcher culturally or theoretically?  Q7. Is the influence of the researcher on the researcher on the research, and vice versa, addressed?  **Group 3: Participants and their voices**  Q8. Are participants, and their voices, adequately represented?  **Group 4: Ethical approval**  Q9. Is the research ethical according to current criteria or, for recent studies, is there evidence of ethical approval by an appropriate body?  **Group 5: Interpretation of the article results**   1. Do the conclusion drawn in the research report flow from the analysis, interpretation, of the data?   **Systematic reviews and research synthesis - Joanna and Briggs Tool**  Q1. Is the review question clearly and explicitly stated?  Q2. Were the inclusion criteria appropriate for the review question?  Q3. Was the search strategy appropriate?  Q4. Were the sources and resources used to search for studies adequate?  Q5. Were the criteria for appraising studies appropriate?  Q6. Was critical appraisal conducted by two or more reviewers independently?  Q7. Were there methods to minimize errors in data extraction?  Q8. Were the methods used to combine studies appropriate?  Q9. Was the likelihood of publication bias assessed?  10. Were recommendations for policy and/or practice supported by the reported data?  Q11. Were the specific directives for new research appropriate?  **Mixed methods appraisal tool (MMAT), version 2018**  Q1. Is there an adequate rationale for a mixed method design to address the research question?  Q2. Are the different components of the study effectively integrated to answer the research question?  Q3. Are the outputs of the integration of qualitative and quantitative components adequately interpreted?  Q4. Are divergence and inconsistencies between quantitative and qualitative results adequately addressed?  Q5. Do the different components of the study adhere to the quality criteria of each tradition of the methods involved?  **Survey-Center for Evidence-Based Medcine (CEBM)**  Q1. Did the study define and address a clearly focused question?  Q2. Is the research method (study design) appropriate for answering the research question?  Q3. Is the method of selecting the subjects (employees, teams, divisions, organizations) clearly described?  Q4. Could the sampling method introduce (selection) bias?  Q5. Is the sample of subjects’ representative of the target population?  Q6. Was the sample size based on pre-study consideration of statistical power considered in advance?  Q7. Was the response rate adequate?  Q8. Are the measurements tool (questionnaires) valid and reliable?  Q9. Was the statistical significance assessed?  Q10. Are confidence intervals given for the main results?  Q11. Could there be confounding factors that haven’t been accounted for?  Q12. Can the results or findings be applied to your organization?  **Narrative - Scale for the Assessment of Narrative Review Articles (SANRA) Tool**  Q1. Is the important of the topic clearly explained and justified?  Q2. Are the reviews aims or specific questions clearly stated?  Q3. Are the literature search strategies described with detail?  Q4. Are key assertions supported by appropriate references?  Q5. Is the argumentation based on appropriate scientific evidence and reasoning?  Q6. Are outcome data presented clearly and appropriately? |
| --- |
