# Supplementary figures and images for: Sociotechnical Cybersecurity Framework for Securing Health Care From Vulnerabilities and Cyberattacks: Scoping Review
Source: J Med Internet Res. 2025 Oct 15;27:e75584. doi: 10.2196/75584 (PMC12572753; doi:10.2196/75584)

Sociotechnical cybersecurity framework implementation steps


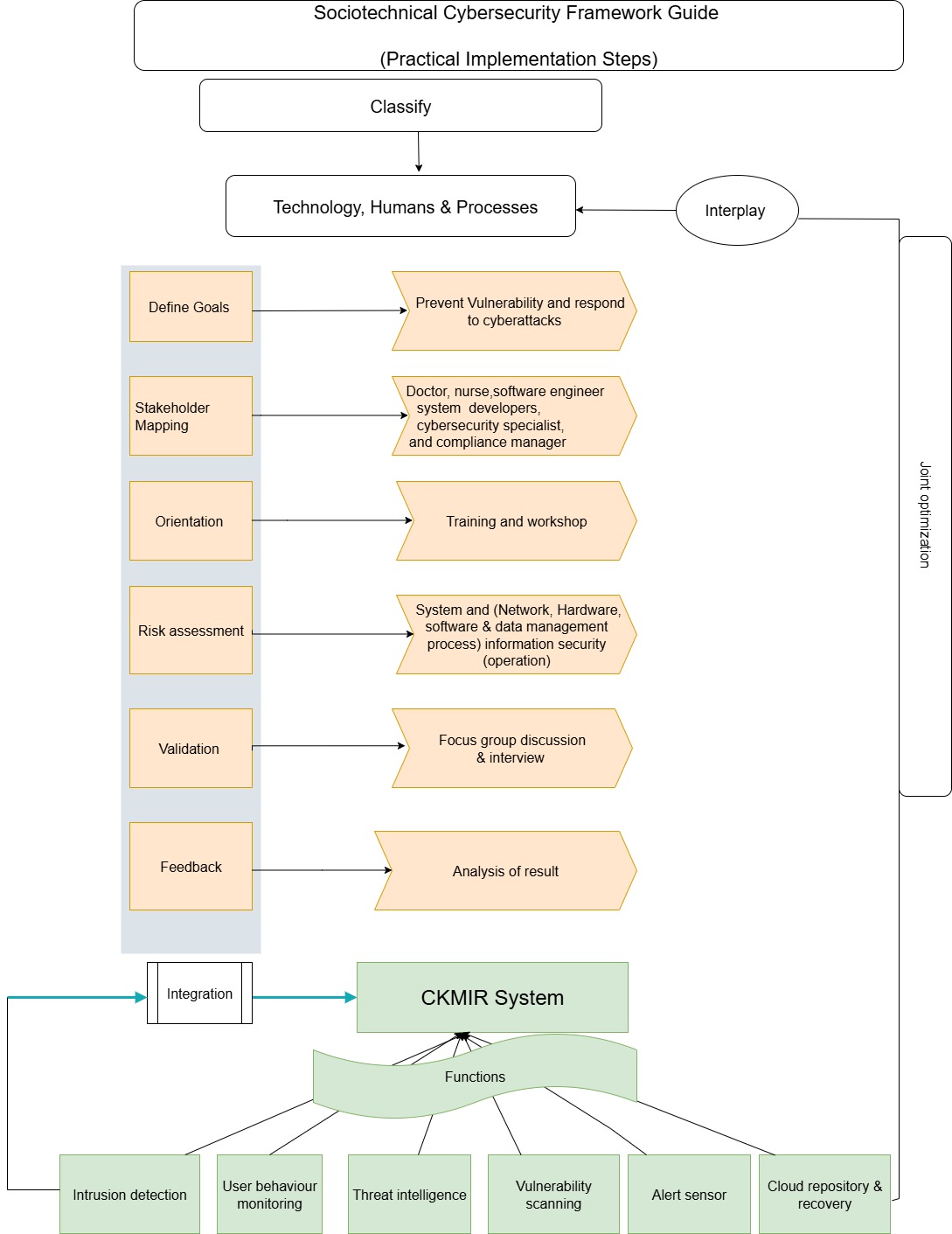

Supplement: Multimedia Appendix 6 [file jmir_v27i1e75584_app6.docx]
