# Supplementary material for: Sociotechnical Cybersecurity Framework for Securing Health Care From Vulnerabilities and Cyberattacks: Scoping Review
Source: J Med Internet Res. 2025 Oct 15;27:e75584. doi: 10.2196/75584 (PMC12572753; doi:10.2196/75584)
Supplement: Multimedia Appendix 7 [file jmir_v27i1e75584_app7.docx]

| **Factors of vulnerability** | **Health care organization action plan** | **Compliance in the health care organization** | **Responsible role** | **Reference** |
| --- | --- | --- | --- | --- |
| Technology |  |  |  |  |
| *Lack of regular audit and assessment* | Regular cybersecurity assessment audits | The health care organization should conduct a monthly and quarterly audit, vulnerability scanning, and assessment to ensure all vulnerabilities are identified  Develop an auditing plan and template | IT team, health care management, compliance officer | Kandasamy et al [45] |
| *New technology integration* | Secure design and technology integration support | Assess security risks before the adoption and integration of new technology  Implement security control for IoT devices and applications across health care systems.  Integrate secure design features in the early stages of development and during new technology integration  Provide self-support and secure guidance | Health care management, procurement manager, original equipment manufacturer (O EM) technology integration expert, compliance officer, IT team | Vukotich [22], Alhammad et al [44]    Badidi et al [126] |
| *Third-party applications and plugins* | Implement third-party application management control | Implement access control and monitor privileges of third-party vendor applications and plugins as they interact with the health care information systems  Health care organizations should implement control applications to monitor and manage third-party technologies, software, applications, and plugins | Procurement manager, OEM, integration expert, compliance officer, IT team, health care management | Arafa et al [42] |
| *Complex system design and usability* | User-centered design | Adopt user-friendly design without compromising security  Promote the adoption of threat modeling in the design approach by incorporating security first into the design plan to meet all legal and regulatory requirements for data privacy and security | System developer, system integrator, health care management, compliance officer, IT team | Badidi et al [126] |
| *Limited monitoring* | Real-time monitoring and alerts | Health care management should engage in continuous monitoring of critical cyber infrastructure, alerting, and automatically counteracting any abnormalities in the network and systems  Continuous updates of health care system devices and applications, policies, and training for emerging threats and trends in health care system | IT team, compliance officer, security operation center (SOC), health care management, human resource team | Sittig and Singh [12], Janith et al [63]    Filipec and Plasilb [52], Abraham et al [61] |
| *Inadequate access control management* | Identity and access control systems | Health care organization should implement strong identity and access control in health information systems to enable audit trail and footprint activities | IT team, human resources team, health management, compliance officer | Wazid et al [53] |
| Human factors |  |  |  |  |
| *Shortage of skilled professionals and limited budget* | Research and development, talent acquisition, increase budget | Health care organizations should partner with educational institutes on research, knowledge transfer, training, and hiring cybersecurity graduates to reduce shortfalls and explore innovative cybersecurity practices and technologies  Increase budget for cybersecurity management. | Human resource team, IT team, health care management, educational institution | DeFord [71], Hines et al [72] |
| *Inefficient training* | Initiate cybersecurity educational training and awareness programs | Management should ensure to provide regular cybersecurity training for all health care staff  Health care management should provide gamification and phishing simulation training tests for employees to check their level of awareness and reinforce cybersecurity awareness training | Human resource team, compliance officer, health care management | Abraham et al [61], Wilner et al [70],  Giansanti and Monoscalco [99] |
| *Insider threats* | Monitoring and behavior analytics | Health care management should implement an organizational cybersecurity culture of inclusiveness, behavioral planning, equal opportunity, and deterrence for all employees  Management should implement cybersecurity access and privileges to restrict employees from unauthorized information  Implement multifactor authentication if data must be accessed in the health care organization  Healthcare should implement clear policies on acceptable use, access control, data handling, and the consequences of violations | Human resource team, compliance officer, IT team, health care management legal policy team, security operation center (SOC) | Wilner et al [70] |
| Security culture | Initiate cybersecurity culture and behavioral measures  Initiate psychological and cultural measures | Health care management and human resources team should conduct behavioral tests to ensure that all employees maintain cyber hygiene  Control over data encryption policy is critical | Human resource team, ethics committees, organizational development team, compliance officer, legal policy team, SOC | Sekandi et al [75], Branley-Bell et al [81]  Yeng et al [76]  Coventry et al [80] |
|  |  | Healthcare organizations should develop guidelines for management to foster a culture of trust, ethical guidance, and transparency for all employees |  |  |
|  |  | Organizational development team should provide a support channel to reduce grievances and manage inappropriate behavior that may lead to insecurity in the workplace |  |  |
| Processes |  |  |  |  |
| *Inadequate policy and procedure* | Collaborative partnership and information sharing policy | Health care organization should partner with cybersecurity experts for support in technology and operational processes to combat challenges  Establish platforms for threat intelligence sharing. Raise awareness of evolving cybersecurity threats and trends for all stakeholders  Regular policy reviews and updating cybersecurity policies should be consistent with regulatory requirements and emerging threats | Human resource team, health care management, compliance officer, support team, IT team, knowledge management consultants | Malatji et al [25], Filipec and Plasilb [52], DeFord [71], Grande et al [95]  Wilner et al [70] |
| *Untimely incident response and recovery plan* | Incident response plan | Develop a contingency and cyber breach incident response and recovery plan in the health care organization  Introduce online and offline cloud-based repositories for restoration of health information in the event of a security incident or ransomware attack in the health care system  The operational manager should develop and establish a communication plan and quick protocol to respond to incidents in the case of cyberthreats and breaches of health information | IT team, compliance officer, SOC, health care management, human resources, computer emergency response team (CERT) | Filipec and Plasilb [52], Abraham et al [61], Wilner et al [70], He et al [87] |
